# Outcomes of home-based versus facility-based care for mild diphtheria during a large epidemic in Kano State, Nigeria: A retrospective matched cohort study

**DOI:** 10.1371/journal.pmed.1005097

**Published:** 2026-10-26

**Authors:** Jonathan Polonsky, Suleiman Hudu, Khadidja Uthman, Yves Katuala, Philip Eghosa Evbuomwan, Hashim Juma Omer Osman, Abdulwahab Kabir Sulaiman, Ichola Ismael Adjaho, Cheick Oumar Doumbia, Etienne Gignoux, Franck Ale

**Affiliations:** 1 Epicentre, Paris, France; 2 Geneva Centre of Humanitarian Studies, Faculty of Medicine, University of Geneva, Geneva, Switzerland; 3 Médecins Sans Frontières, Operational Centre West and Central Africa (MSF WaCA), Abidjan, Côte d’Ivoire; 4 Kano State Ministry of Health, Kano, Nigeria; Duke University, UNITED STATES OF AMERICA

## Abstract

**Background:**

During Nigeria’s largest recorded diphtheria epidemic (first notified on 1 December 2022; more than 40,000 confirmed cases by January 2026), hospital capacity in Kano State was overwhelmed. Médecins Sans Frontières (MSF) introduced home-based care (HBC) for patients with mild disease; we asked whether mortality, long-term sequelae, and household transmission differed between HBC and care in a diphtheria treatment centre (DTC).

**Methods and findings:**

We conducted a retrospective matched cohort study using MSF registry data and household interviews to compare mortality, long-term sequelae, and household transmission between patients with mild diphtheria treated under HBC or in a DTC. The primary outcomes were mortality, long-term sequelae, and household transmission. Mild diphtheria cases notified between January 2023 and January 2024 were exactly matched by treatment modality (HBC or DTC) on sex, age group, vaccination status, and ward of residence, and interviewed between September and October 2024, a median of 421 days after notification. Conditional logistic regression, adjusted for age, vaccination status, and treatment delay, was used to estimate the association between treatment modality and mortality, with robustness checked with propensity score weighting and sensitivity analyses. Of the 986 originally sampled patients, 491 (49.8%) were interviewed; with 178 replacements (substitute patients of similar age, sex, vaccination status, and area, interviewed when an originally selected patient could not be), 669 patients (362 HBC, 307 DTC) were analysed. After adjustment, mortality was lower with HBC than DTC care, but the interval included 1 (odds ratio [OR] 0.36, 95% confidence interval [CI] [0.12,1.07]; *p* = 0.066); treatment delay was associated with increased mortality (OR 1.29 per day, 95% CI [1.00,1.67]; *p* = 0.048). Adding calendar time gave a similar estimate (OR 0.35, 95% CI [0.08,1.57]; *p* = 0.170). Vaccination was associated with lower odds of death (OR 0.32, 95% CI [0.10,0.98]; *p* = 0.047). HBC was not associated with increased household transmission or long-term sequelae. Residual confounding by disease severity, the small number of deaths (24), and replacement patients limit these findings.

**Conclusions:**

Vaccination and early treatment were associated with survival; care setting was not clearly associated. When supported by clinical triage and structured follow-up, decentralised care may be an option to manage mild cases during diphtheria epidemics in settings with constrained hospital capacity.

## Author summary

**Why was this study done?:**

- Diphtheria epidemics have re-emerged across West and Central Africa, and during large outbreaks, hospital treatment capacity can be overwhelmed.
- During a 2023–2024 epidemic in Kano State, Nigeria, home-based care was introduced for patients with mild diphtheria to relieve pressure on treatment centres, but little evidence existed on how its outcomes compared with facility-based care.

**What did the researchers do and find?:**

- The researchers compared outcomes for 669 patients with mild diphtheria, matched on age, sex, vaccination status, and area of residence, who were treated either at home or in a diphtheria treatment centre.
- Home-based care was not associated with increased mortality, household transmission, or long-term complications, and patients reported high acceptability for both approaches.
- Survival was associated with how quickly treatment began and with prior vaccination status.

**What do these findings mean?:**

- Carefully structured home-based care, with reliable triage, prompt antibiotic treatment, and a referral pathway for patients who worsen, may be an option to extend treatment capacity during diphtheria epidemics in settings with limited hospital resources.
- Because the study compared groups that were not randomly assigned to each type of care, and included only patients already judged to have mild disease, doctors making more complex triage decisions may have unintentionally influenced the comparison, and a study that randomly assigns patients to each type of care would give more confidence before home-based care is used more widely.

## Abstract 3

In a cohort study, Jonathan Polonsky and colleagues compare outcomes of home-based and facility-based care during a major diphtheria outbreak in Nigeria, finding that home-based care was not associated with increased mortality or transmission, and could be an option for mild disease when facility capacity is overwhelmed.

## Introduction

Conventional management of diphtheria requires hospitalisation for administration of diphtheria antitoxin (DAT), antibiotic therapy, and monitoring for toxin-mediated complications such as myocarditis and polyneuropathy [1,2]. This assumes the availability of inpatient capacity, trained staff, and a reliable DAT supply, conditions rarely met during large epidemics in resource-constrained settings. In Bangladesh, nine dedicated diphtheria treatment centres (DTCs) struggled to meet demand during the 2017–2019 Rohingya refugee epidemic despite substantial international support [3]. During the same epidemic, DAT shortages forced clinicians to ration treatment [3]. When the current epidemic struck Kano State, Nigeria, in late 2022 (first notified on 1 December 2022), hospital capacity was overwhelmed within months [4,5].

Nigeria’s ongoing diphtheria epidemic is the largest in Africa in decades [4], with more than 40,000 confirmed cases nationally and 24,687 confirmed cases in Kano State alone by January 2026 [6]. A companion population-based survey estimated that community attack rates in the most affected areas were approximately three times the rate notified at MSF-supported treatment sites, with the majority of deaths occurring at home [7]. Facility-based data from Kano documented a case fatality rate of 4.5% among 18,320 surveillance-captured cases [4].

In response to rapidly increasing cases during mid-2023, Médecins Sans Frontières (MSF), in collaboration with the Kano State Ministry of Health, introduced home-based care (HBC) for patients assessed as having mild diphtheria without signs of complications. These patients received oral antibiotics and were discharged home with follow-up visits on days 3 and 7, weekly outpatient reassessment, and a clear referral pathway to a DTC if they deteriorated. Patients with signs of severe disease (respiratory distress, extensive pharyngeal membrane, or suspected toxin-mediated complications) were admitted to DTCs for inpatient management, including DAT. Over the 15-month response period, MSF managed 14,707 patients, of whom 6,696 (45.5%) received HBC.

Severity and complication risk in diphtheria are determined largely by the extent and duration of toxin exposure [1,2]. Patients with mild pharyngeal disease and limited pseudomembrane who receive early antibiotics to halt toxin production might plausibly be managed outside hospital. However, the risk of clinical deterioration (particularly late-onset myocarditis, which can occur days to weeks after initial presentation) and the potential for increased household transmission represent key concerns [2,8].

We conducted a retrospective matched cohort study to ask whether mortality, long-term sequelae, and household transmission differed between HBC and DTC-based care for mild diphtheria during the Kano State epidemic. We also assessed the secondary outcome of patient-reported acceptability, and examined clinical and care-delivery factors associated with these outcomes.

## Methods

### Study design and setting

This was a retrospective matched cohort study of patients with mild diphtheria recorded in Médecins Sans Frontières (MSF) registries in four Local Government Areas (LGAs) of Kano State: Dawakin Tofa, Fagge, Nassarawa, and Ungogo. Patients were either admitted to a DTC at one of two hospitals, the Infectious Diseases Hospital and the Murtala Muhammed Specialist Hospital, or treated at home through HBC, which MSF introduced for mild disease in July 2023. Patients with mild disease in both arms were treated with antibiotics and did not routinely receive DAT. A third site, Waziri Shehu Gidado General Hospital, contributed only HBC patients to the sample. We interviewed patients or their caregivers between 18 September and 21 October 2024. This study is reported according to the Strengthening the Reporting of Observational Studies in Epidemiology (STROBE) guidelines (S1 Checklist).

### Protocol and analysis plan

The study followed a protocol (version 2024-07-08) that the MSF Ethics Review Board approved on 9 July 2024 (reference 2437), before data collection began (S1 File), with a table of departures from it (S2 File). It specified the retrospective matched cohort design, the comparison of death or long-term sequelae and of household secondary attack rates between HBC and DTC care, and the sample size. The protocol stated no formal hypothesis; its objective was to evaluate the performance of HBC in terms of treatment outcome and household secondary attack rates, and all comparisons in this paper are two-sided. Its analysis section described descriptive statistics and simple tests, and did not specify the models used to compare HBC with DTC care. The primary conditional logistic regression, its covariates, and every sensitivity analysis were therefore developed during data analysis. Analyses added in response to peer review are identified as post hoc in the sections below.

### Participants and matching

Eligible patients were those recorded in the MSF line list with mild diphtheria at initial assessment, notified between 1 January 2023 and 31 May 2024 at one of the three study hospitals, lived in one of the four LGAs, had not been readmitted, and had a unique 10-digit telephone number, which were needed to arrange the interview. This gave a sampling frame of 3,462 patients (968 DTC and 2,494 HBC); the sampled patients were notified between 12 January 2023 and 23 January 2024. We defined strata by age group (0–5, 6–10, 11–15, and 16 years or more), sex, ward of residence, and vaccination status as recorded in the registry, and sampled only from strata that contained both DTC and HBC patients. We selected 496 DTC patients, allocating the number per LGA in proportion to the eligible patients living there and choosing patients within each LGA at random with a probability proportional to the number of DTC-HBC pairs available in their stratum. For each stratum we then drew at random as many HBC patients as we had sampled DTC patients. Four strata contained fewer eligible HBC than sampled DTC patients, and we sampled all HBC patients in them. This yielded 490 HBC patients and 986 sampled patients in total, rather than the full target of 992. Matching was exact, no patient was sampled more than once, and we applied no calliper because all matching variables were categorical.

Field teams telephoned each sampled patient or household to confirm identity, obtain agreement, and arrange a home visit, and made two contact attempts by telephone before recording the outcome. A patient who could not be reached after two attempts, declined, or was not interviewed at the arranged visit was replaced with the help of a study supervisor, meaning that we interviewed a substitute patient from the same registry, who had not been selected for the study but resembled the original patient in age group, sex, vaccination status, and area of residence. Candidates for replacement were unsampled patients from the same stratum and treatment arm. When a stratum and arm had no candidates remaining, we drew from another stratum with the same age group, sex, vaccination status, and LGA (and therefore a different ward) and the same treatment arm, and, for the strata still depleted, from such a stratum of either arm. Replacements were assigned to strata, not to named original patients, and were analysed in the stratum they came from. The approved protocol provided for exclusion after two unsuccessful attempts and made no provision for replacement (S2 File).

Before MSF introduced HBC in July 2023, patients with mild diphtheria were generally admitted to a DTC. From July 2023, patients without respiratory distress and with limited pseudomembrane extension were eligible for HBC, and patients with signs of moderate or severe disease were admitted. Among eligible patients, the treatment setting also depended on clinician judgement, the suitability of the household, site and bed capacity, and the preference of the patient or caregiver. Treatment setting was therefore associated with the date of presentation.

### Data collection

Trained interviewers visited each patient (or their caregiver) at home and administered a structured questionnaire using KoboCollect [9] on encrypted tablets. The questionnaire (S3 File) recorded demographic characteristics, vaccination status, dates of symptom onset and treatment, the outcome of the illness, long-term sequelae (assessed at the time of interview), and diphtheria status among household members. A dedicated section assessed patient-reported acceptability using a five-point Likert scale across three dimensions: perceived improvement, acceptability of the care model, and perceived quality. The MSF registry line list supplied the variables used for sampling and matching (age group, sex, ward of residence, and vaccination status), together with admission location, date of notification, and clinical complications. Age, sex, and vaccination status were asked again at interview. Matching used the registry values, and the analyses used the interview values, with registry values substituted only where the interview response was missing.

### Definitions

A diphtheria case was any patient recorded in the MSF registry with a clinical diagnosis of suspected diphtheria, regardless of laboratory confirmation. Mild disease was defined by the absence of respiratory distress and limited pseudomembrane extension on clinical examination at presentation. Vaccination status was the respondent’s report at interview of receipt of at least one dose of a diphtheria-containing vaccine, with the registry dose count used where the respondent did not know. Two alternative definitions were used in sensitivity analyses (post hoc). The first counted a patient as vaccinated when the report was supported by a vaccination card, by a card reported lost, or by vaccination at the treatment facility, and as unvaccinated when no vaccination was reported or when vaccination was reported without a card or facility record; patients with unknown card status were excluded. The second compared patients whose vaccination was confirmed by a card with patients who reported no vaccination, and excluded all others. Treatment delay was the number of days from symptom onset to the start of treatment, as reported at interview.

All-cause mortality was death from any cause between presentation and the interview, as reported by the respondent. Long-term sequelae were any sequela reported by the respondent at the interview, assessed among survivors, and therefore reflect status at that single time point. Clinical complications were serious events recorded by clinicians in the registry line list: acute kidney injury, thrombocytopenia, airway obstruction, heart failure, and neurological involvement. Secondary household cases were household members who developed diphtheria-compatible symptoms 2–14 days after the index patient’s symptom onset. Sensitivity analyses used a window of 2–30 days and an unrestricted window.

### Outcomes

All-cause mortality was the principal primary outcome. Long-term sequelae among survivors and the intra-household secondary attack rate (SAR) were the other primary outcomes. Patient-reported acceptability was a secondary outcome. A composite of death or long-term sequelae was analysed as a supplementary outcome (post hoc).

### Sample size

The protocol set a target of 496 patients per group (992 in total) for a comparison of the proportion with death or long-term sequelae between DTC and HBC care. It assumed a case fatality risk of 2.5% in the DTC group and 5% in the HBC group, a two-sided alpha of 0.05, 80% power, and 10% non-response. A repeat calculation with these assumptions gives about 905 patients per group before allowance for non-response, and about 55% power at 496 per group. We interviewed 669 patients (307 DTC, 362 HBC).

### Statistical analysis

The primary estimand was the conditional odds ratio (OR) for death comparing HBC with DTC care within matched strata. We estimated it with conditional logistic regression stratified by matched subclass, so that the estimate rests on within-subclass comparisons.

Each subclass contained all patients sharing the same age group, sex, ward of residence, and registry vaccination status, so subclass size varied from 1 to 39 patients, and subclasses were not fixed 1:1 pairs. Of the 143 subclasses, 57 (72 patients) contained one arm only and did not contribute to the conditional estimate. Case fatality risks and unadjusted and adjusted risk ratios (RRs) were estimated using a design-based approach (R package survey) that treated each subclass as a stratum (post hoc for the RRs). These RRs are population-averaged: they do not condition on matched sets, and they include the patients in single-arm subclasses. We did not adjust for multiple comparisons.

Analyses were conducted in R (version 4.5.2). The packages and model specifications are listed in S1 Text. Proportions are presented with 95% confidence intervals (CIs): design-based intervals for case fatality risks and exact binomial intervals for SARs. The number of complete cases and events in each model is reported in the Results and in S1 Table.

The primary model included treatment modality, age, vaccination status, and treatment delay as covariates. Age was modelled continuously in years and treatment delay continuously in days, so that odds ratios for these terms are per one-year and per one-day increase. To assess departure from linearity in treatment delay, we compared the linear specification with a restricted cubic spline (4 knots) using a likelihood ratio test, and retained the linear specification unless the spline improved fit significantly. Clinical complications were excluded from the primary model because they occurred after treatment allocation and could mediate part of the association between treatment setting and mortality.

Diagnostics for the primary model (post hoc) are reported in S2 Table. The conditional likelihood has no intercept, so its fitted values have no absolute scale. We therefore assessed collinearity (variance inflation factors), discrimination (c-statistic), and calibration (observed against predicted risk by quintile) in an unconditional logistic regression with the same covariates, and additionally report the within-subclass concordance of the conditional model. Case-level influence was assessed with dfbeta residuals from a refit of the primary model using the Efron method, because dfbeta residuals are not available for the exact method.

Calendar time was assessed as a potential confounder, because HBC was introduced part-way through the epidemic and care-seeking and system capacity may have changed over time. We added a restricted cubic spline (4 knots) of the date of notification to the primary model and compared it with the model without calendar time using a likelihood ratio test. Calendar time was to be retained in the primary model only if the test indicated a significant improvement in fit. Because treatment arm and calendar time were closely related, we report the calendar-adjusted estimate alongside the primary estimate regardless of the test result (post hoc). We also tested whether the association between treatment modality and mortality differed between an early and a late phase of the epidemic, split at the median notification date, using an interaction term.

Ward of residence was an exact-matching variable and each ward lies within a single LGA, so every matched subclass sits within one LGA and the conditional model cannot estimate an LGA effect. In two post hoc checks, we added LGA as a fixed covariate to the primary model (which leaves the treatment estimate unchanged by construction) and fitted an unconditional logistic regression with the same covariates and a random intercept for LGA. The second model ignores the matched subclasses and models variation between LGAs explicitly; we treated it as a supportive check and not as an alternative primary analysis.

Long-term sequelae were analysed among survivors. We used conditional logistic regression, stratified by matched subclass, with treatment modality, age, vaccination status, and treatment delay as covariates, as in the primary mortality model. Clinical complications were not included because they occurred after allocation. Because there were few events, we also fitted the conditional model with treatment modality alone, Firth penalised logistic regression with the primary covariates and with complications added (post hoc), the conditional model restricted to survivors without complications (post hoc), and design-based RRs and risk differences with subclass as the stratum (post hoc). The Firth models do not condition on matched subclasses, and the design-based estimates are population-averaged and include patients in single-arm subclasses.

Household transmission was analysed using the household roster that respondents reported at the index patient’s interview. The SAR was the number of secondary cases divided by the number of household members listed. The primary case-ascertainment window was 2–14 days after the index patient’s symptom onset, following the typical incubation period of 2–5 days: household members whose onset came sooner than this were unlikely to have been infected by the index patient, and the upper bound allows for later onset. We examined risk factors for secondary infection within the primary window using logistic regression with standard errors clustered by household. Covariates were the index patient’s treatment modality, clinical complications, treatment delay (4 days or more), and vaccination status; household size; and the household member’s age group, sex, and vaccination status. Household members with missing covariates, and those who became cases outside the window, were excluded from these models. Because intra-household transmission was not rare, we also estimated RRs from Poisson regression with cluster-robust standard errors and the same covariates (post hoc; S3 Table), and repeated the logistic model in the two wider windows. As a check on within-household correlation, we fitted a logistic model with a household-level random intercept (post hoc), omitting the member’s age group and sex because the full model was singular.

Acceptability was analysed using linear mixed-effects models with a random intercept for matched subclass, fitted separately for each Likert dimension and for a composite score, the mean of the three dimension scores. Each model included treatment modality, treatment delay (4 days or more), and clinical complications. Coefficients are mean differences in Likert points, HBC minus DTC, and are not transformed. Because the scores are ordinal, we also fitted a cumulative-link (proportional-odds) regression to each dimension with the same covariates (post hoc; S6 Table). These models omit the subclass random intercept, which could not be estimated: a cumulative-link mixed model gave a subclass standard deviation close to zero and an ill-conditioned Hessian for all three dimensions. To account for the matched design, we obtained 95% CIs for the treatment and delay coefficients by resampling subclasses with replacement (2,000 replicates, percentile intervals; post hoc). Replicates in which the model did not meet convergence criteria were excluded. The complication coefficient was not bootstrapped because it was unstable in resamples with few complicated respondents.

### Sensitivity and robustness analyses

We conducted sensitivity analyses of the primary mortality model. First, we added clinical complications to the primary model (mediator-adjusted model). Second, we excluded patients with clinical complications and refitted the primary model. Third, because the primary model had few events relative to covariates (24 deaths and four covariates), we fitted a Firth penalised logistic regression (post hoc) with treatment modality, age, vaccination status, and treatment delay as covariates; this model does not condition on matched subclasses. Fourth, we refitted the primary model with each of the two card-based definitions of vaccination status (post hoc), keeping the matched subclasses defined by registry vaccination status. Fifth, we tested for effect modification by treatment delay, adding a treatment-by-delay interaction (delay in days) to the primary model and comparing the two models with a likelihood ratio test (post hoc). Sixth, we fitted the primary model to the composite of death or long-term sequelae (post hoc), with a Firth penalised model as a check. Finally, we calculated *E*-values for the treatment modality, vaccination, and treatment delay estimates of the primary model, treating death as a rare outcome, to quantify the minimum strength of association that an unmeasured confounder would need with both treatment and death to explain each estimate [10].

As a complementary estimator that does not rely on the matched subclasses, we estimated the marginal association between treatment modality and mortality with stabilised inverse probability of treatment weighting (IPTW) in the 669 patients in the primary analysis. The estimand was the average treatment effect in these patients, including those in single-arm subclasses. Weights were derived from a logistic regression of treatment allocation on age, age squared, sex, vaccination status, LGA of residence, and household size, and were trimmed at the 1st and 99th percentiles to limit the influence of extreme weights. The marginal odds ratio came from a weighted logistic regression of death on treatment modality alone, and a doubly robust odds ratio (post hoc) from the same model with age, vaccination status, and treatment delay added. We also estimated the marginal RR and risk difference from weighted quasi-Poisson and linear regressions (post hoc). Standard errors were heteroscedasticity-robust (HC1) and treat the estimated propensity scores as fixed, so they do not reflect uncertainty in estimating the weights. We assessed positivity from the distribution and range of propensity scores in each group and the proportion of scores below 0.05 or above 0.95, and covariate balance after weighting from standardised mean differences (post hoc). We also compared the primary trimming threshold with no trimming and with trimming at the 5th and 95th percentiles (post hoc). The propensity score range, covariate balance, effective sample sizes, and trimming results are reported in S4 Table.

To assess whether patients we interviewed differed from those we did not, we compared the originally sampled patients, interviewed or not interviewed, on the registry variables available for all of them (sex, age group, vaccination status, and admission location) using chi-squared tests. As a selection-weighted sensitivity analysis (post hoc), we multiplied the IPTW weights by inverse probability of interview weights. These came from a logistic regression of interview status among the originally sampled patients on the same four registry variables, stabilised by the marginal probability of interview. Replacement patients were given the probability predicted from their own registry values, on the assumption that their probability of interview resembles that of original patients with the same values; this assumption cannot be verified. We estimated the marginal odds ratio from a weighted logistic regression of death on treatment modality, with and without age, vaccination status, and treatment delay. In a further sensitivity analysis (post hoc), we restricted the primary model, a Firth penalised logistic regression, and IPTW to the originally sampled patients who were interviewed, re-estimating the propensity model within this subset. Multiple imputation was not used: patients who were not interviewed had no outcome data, and only these four registry variables were available for imputation.

### Ethics

The MSF Ethics Review Board approved the protocol (version 2024-07-08; ID 2437) on 9 July 2024, and the Kano State Health Research Ethics Committee approved the study (reference SHREC/2024/5164, letter dated 13 June 2024; NHREC registration number NHREC/17/03//2018). Before the study began, the study team met the community leaders of the study wards, explained the study, and obtained their oral approval and support. Written informed consent was obtained from all participants or their guardians, using signature or, for illiterate participants, fingerprint; for illiterate participants, an independent witness chosen by the participant and unconnected to the research team attested that the information was accurately explained and that consent was given freely. Data were anonymised and stored in accordance with data protection regulations.

### Inclusivity in global research

Additional information regarding the ethical, cultural, and scientific considerations specific to inclusivity in global research is included in the Supporting Information (S2 Checklist).

### Use of AI Disclosure

Two generative artificial intelligence (AI) tools were used. The MSF AI Assistant (ai.msf.org; powered by GPT-5.2 via Microsoft Azure OpenAI) was used to assist with the development and debugging of selected R analysis code and to revise selected manuscript text for clarity and concision. During the revision of this manuscript, Claude (Anthropic; Claude Code, Sonnet 5.5 and Opus 5.5) was used to assist with revising and checking the R analysis code, re-running the analyses, preparing the supporting information files, and drafting and editing the manuscript text. All AI-assisted code was reviewed by the authors and executed within the study analysis environment. The analysis pipeline was re-run in full from the study data after code changes, and analytical outputs were checked against the source data and the study’s intended methods before inclusion in the manuscript. The analysis code is publicly available (see Data sharing). All AI-assisted manuscript text was reviewed and edited by the authors. The authors take full responsibility for the analytical methods used, the accuracy of the analyses and manuscript, and all interpretations, results, and conclusions presented.

## Results

### Participants

Of the 986 sampled patients (496 DTC and 490 HBC), 491 (49.8%) were interviewed: 250/496 DTC patients (50.4%) and 241/490 HBC patients (49.2%) (Fig 1). Of the 986, 559 (56.7%) were reached by telephone within two attempts (278/496 DTC patients, 56.0%, and 281/490 HBC patients, 57.3%), and 491 of these 559 (87.8%) were interviewed (250/278, 89.9%, in the DTC arm and 241/281, 85.8%, in the HBC arm). The 495 sampled patients who were not interviewed comprised 427 who could not be reached after two attempts, 51 who agreed by telephone but were absent at the arranged visit, 12 who refused by telephone, and 5 who refused at the visit. A further 178 patients were interviewed as replacements (57 DTC and 121 HBC), and one replacement refused at the visit. The analysis population was therefore 669 interviewed patients (307 DTC and 362 HBC), all of whom were included in the primary model (S1 Table), although the 72 in single-arm subclasses do not contribute to the conditional estimate.

**Fig 1:**
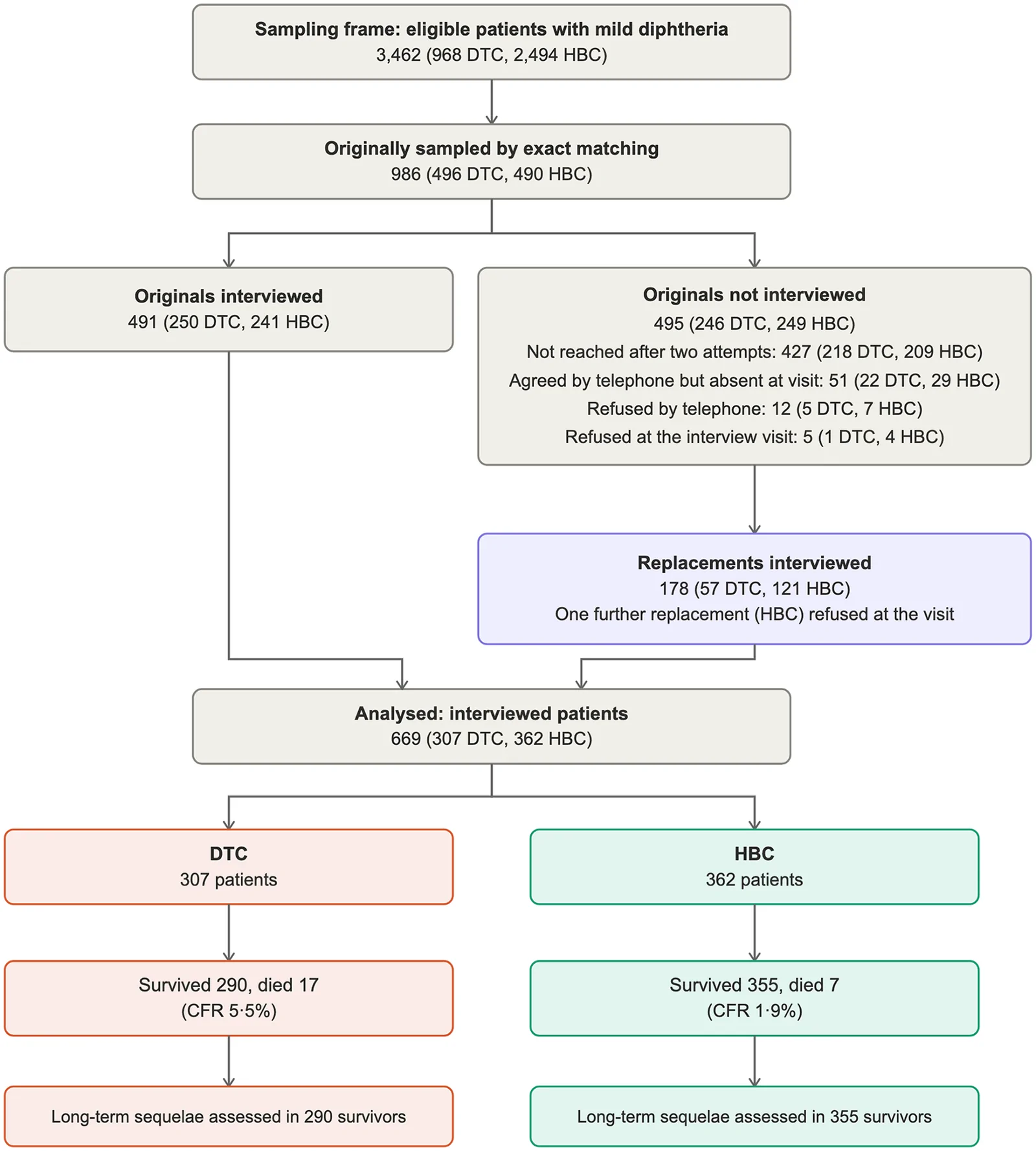
Flow of participants from the MSF case registry through sampling, interview, and analysis, by treatment modality, Kano State diphtheria epidemic, Nigeria, 2023–2024. MSF, Médecins Sans Frontières; HBC, home-based care; DTC, diphtheria treatment centre; CFR, case fatality risk.

Replacements made up 178/669 (26.6%) of interviewed patients: 121/362 (33.4%) in the HBC arm and 57/307 (18.6%) in the DTC arm. Replacements were assigned to strata and not to named patients, so the arm of the patient replaced is known only where an original patient was recorded against the replacement. This applied to 85 replacements, of whom 8 (9.4%) were treated in the other arm: 5 DTC patients were replaced by HBC patients and 3 HBC patients by DTC patients. For the other 93 replacements the patient replaced was not recorded, so the number that crossed arm is not known.

Among the 986 originally sampled patients, those interviewed (*n* = 491) and those not interviewed (*n* = 495) differed by sex (males 180/491, 36.7%, versus 148/495, 29.9%; *p* = 0.029) and by vaccination status at registration (fully vaccinated 115/491, 23.4%, versus 93/495, 18.8%; *p* = 0.043), but not by age group (*p* = 0.136) or treatment arm (*p* = 0.750) (S5 Table). In the 669 interviewed patients, who include the replacements, the absolute standardised mean differences between arms were at most 0.104 for the matching variables (age group, sex, vaccination status at registration, and LGA) (S5 Table). The variables that were not matched on were less balanced: 0.19 for household size, 0.18 for comorbidity, and 0.23 for clinical complications.

Among the 669 interviewed patients, the median date of notification was 3 July 2023 (interquartile range [IQR] [9 April,11 August]) for DTC patients and 3 September 2023 (IQR [13 August,4 October]) for HBC patients (Fig 2). No HBC patient was notified before 1 July 2023, compared with 149/307 (48.5%) of DTC patients.

**Fig 2:**
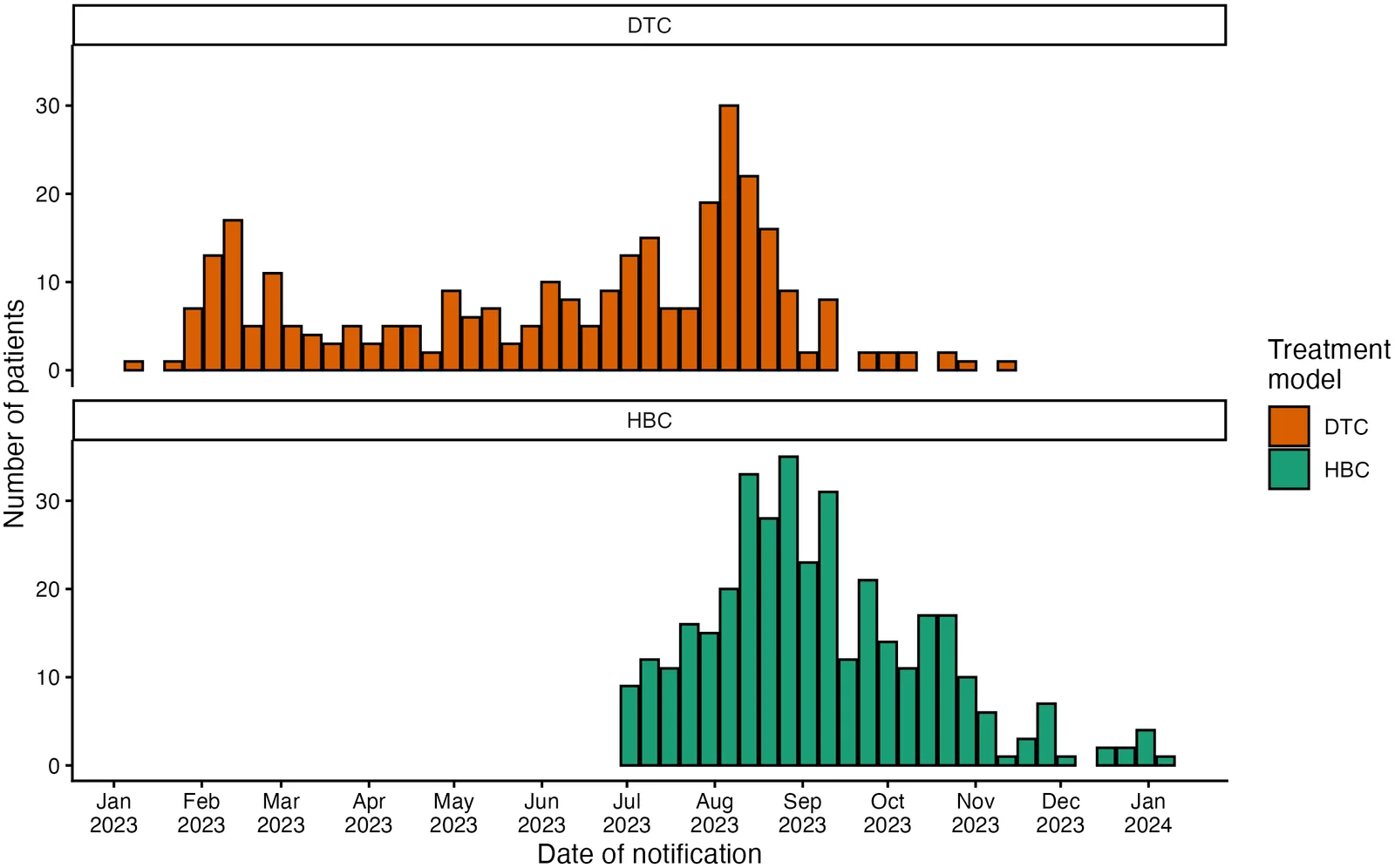
Epidemic curve of enrolled participants by week of notification and treatment modality, Kano State diphtheria epidemic, Nigeria, 2023–2024. HBC, home-based care; DTC, diphtheria treatment centre.

The median age was 11 years (IQR [8,16]) and 418/669 (62.5%) were female, with similar age and sex distributions in the two arms (Table 1). Vaccination coverage was 447/669 (66.8%; 95% CI [63.4,70.2]): 212/307 (69.1%) in the DTC arm and 235/362 (64.9%) in the HBC arm. Five DTC patients (1.6%) and no HBC patients had a documented comorbidity (peptic ulcer disease in four and diabetes in one), and eight DTC patients (2.6%) and no HBC patients had a clinical complication (acute kidney injury in four, thrombocytopenia in three, airway obstruction in two, heart failure in two, and peripheral neuritis and nasal voice in one each; four patients had more than one type). Median household size was 8 (IQR [5,10]) in the DTC arm and 7 (IQR [5,9]) in the HBC arm (Wilcoxon rank-sum test, p = 0.015), and median treatment delay was 2 days (IQR [1,3]) in both arms (*p* = 0.436).

**Table 1:** Characteristics of enrolled participants by treatment modality, Kano State diphtheria epidemic, Nigeria, 2023–2024.

Characteristic	HBC (*n* = 362)	DTC (*n* = 307)	Total (*n* = 669)
LGA			
Dawakin Tofa	20 (5.5%)	11 (3.6%)	31 (4.6%)
Fagge	106 (29.3%)	86 (28.0%)	192 (28.7%)
Nassarawa	45 (12.4%)	39 (12.7%)	84 (12.6%)
Ungogo	191 (52.8%)	171 (55.7%)	362 (54.1%)
Sex			
Female	226 (62.4%)	192 (62.5%)	418 (62.5%)
Male	136 (37.6%)	115 (37.5%)	251 (37.5%)
Age group			
0–5 years	37 (10.2%)	35 (11.4%)	72 (10.8%)
6–10 years	137 (37.8%)	110 (35.8%)	247 (36.9%)
11–15 years	99 (27.3%)	79 (25.7%)	178 (26.6%)
≥16 years	89 (24.6%)	83 (27.0%)	172 (25.7%)
Vaccinated			
No	127 (35.1%)	95 (30.9%)	222 (33.2%)
Yes	235 (64.9%)	212 (69.1%)	447 (66.8%)
Comorbidity	0	5 (1.6%)	5 (0.7%)
Complication	0	8 (2.6%)	8 (1.2%)
Median household size (IQR)	7 [5,9]	8 [5,10]	7 [5,10]
Median treatment delay, days (IQR)	2 [1,3]	2 [1,3]	2 [1,3]
Outcome			
Recovered	349 (96.4%)	282 (91.9%)	631 (94.3%)
Died	7 (1.9%)	17 (5.5%)	24 (3.6%)
Still unwell	6 (1.7%)	8 (2.6%)	14 (2.1%)

HBC, home-based care; DTC, diphtheria treatment centre; LGA, local government area; IQR, interquartile range. Percentages are column percentages.

### Mortality

There were 24 deaths among 669 interviewed patients (case fatality risk (CFR) 3.6%, 95% CI [2.2,5.0]): 7 among 362 HBC patients (1.9%, 95% CI [0.5,3.4]) and 17 among 307 DTC patients (5.5%, 95% CI [3.0,8.1]) (Table 1). The unadjusted RR for death with HBC relative to DTC was 0.35 (95% CI [0.15,0.83]; *p* = 0.017), and the unadjusted conditional OR within matched subclasses was 0.32 (95% CI [0.12,0.87]; *p* = 0.025) (Table 2). Case fatality was highest among patients aged 0–5 years (8/72, 11.1%), compared with 8/247 (3.2%) at 6–10 years, 7/178 (3.9%) aged 11–15 years, and 1/172 (0.6%) at 16 years or older.

**Table 2:** Conditional logistic regression of predictors of mortality among interviewed participants, Kano State diphtheria epidemic, Nigeria, 2023–2024, with unadjusted, primary (Model A), mediator-adjusted sensitivity (Model B), and calendar-time sensitivity estimates shown side by side.

Predictor	Unadjusted OR (95% CI; *p*)	Model A OR (95% CI; *p*)^*^	Model B OR (95% CI; *p*)^†^	Model A plus calendar time OR (95% CI; *p*)^‡^
Treatment: HBC vs. DTC	0.32 ([0.12,0.87]; *p* = 0.025)	0.36 ([0.12,1.07]; *p* = 0.066)	0.47 ([0.15,1.49]; *p* = 0.202)	0.35 ([0.08,1.57]; *p* = 0.170)
Age, per year increase	0.75 ([0.59,0.95]; *p* = 0.018)	0.78 ([0.61,0.99]; *p* = 0.044)	0.74 ([0.56,0.96]; *p* = 0.024)	0.77 ([0.59,0.99]; *p* = 0.045)
Vaccinated vs. unvaccinated	0.24 ([0.08,0.71]; *p* = 0.010)	0.32 ([0.10,0.98]; *p* = 0.047)	0.27 ([0.08,0.91]; *p* = 0.034)	0.32 ([0.10,1.00]; *p* = 0.050)
Treatment delay, per day	1.21 ([0.97,1.50]; *p* = 0.085)	1.29 ([1.00,1.67]; *p* = 0.048)	1.26 ([0.94,1.69]; *p* = 0.123)	1.32 ([1.01,1.72]; *p* = 0.042)
Complication present vs. absent	15.7 ([2.22,111]; *p* = 0.006)	Not included	22.4 ([1.77,283]; *p* = 0.016)	Not included

HBC, home-based care; DTC, diphtheria treatment centre; OR, odds ratio; CI, confidence interval.

*Model A (primary, *n* = 669, 24 deaths): died ~ treatment modality + age + vaccination status + treatment delay, stratified by matched subclass; excludes clinical complications (analysed as an intermediate outcome rather than a baseline confounder).

†Model B (*n* = 669, 24 deaths): as Model A, a sensitivity analysis additionally adjusting for clinical complications.

‡Model A plus a restricted cubic spline (4 knots, 3 degrees of freedom) of the date of notification (*n* = 669, 24 deaths); the calendar-time spline is summarised by the joint likelihood ratio test (3 degrees of freedom, *p* = 0.895). ORs in Models A and B are adjusted for the other covariates shown. Unadjusted estimates use the same matched-subclass-stratified conditional logistic regression, variable definitions, and *n* = 669 analytic sample as Models A and B.

In the primary model, adjusted for age, vaccination status, and treatment delay, the odds of death were lower with HBC than with DTC care, but the interval included 1 (OR 0.36, 95% CI [0.12,1.07]; *p* = 0.066) (Table 2). The corresponding population-averaged RR was 0.36 (95% CI [0.16,0.83]; *p* = 0.016). Each additional day of treatment delay was associated with 29% higher odds of death (OR 1.29 per day, 95% CI [1.00,1.67]; *p* = 0.048). A restricted cubic spline for delay did not significantly improve fit over the linear term (likelihood ratio test, *χ*^2^ = 5.62, 2 degrees of freedom, *p* = 0.060) and the linear term was retained. Vaccination was associated with lower odds of death (OR 0.32, 95% CI [0.10,0.98]; *p* = 0.047), as was each additional year of age (OR 0.78, 95% CI [0.61,0.99]; *p* = 0.044). The vaccination odds ratio was 0.23 (95% CI [0.06,0.84]; *p* = 0.026) with the first card-based definition (648 patients, 24 deaths) and 0.44 (95% CI [0.05,4.06]; *p* = 0.466) with the second (275 patients, 17 deaths).

When clinical complications were added to the primary model (mediator-adjusted model), the estimate for HBC moved towards 1 (OR 0.47, 95% CI [0.15,1.49]; *p* = 0.202), and complications were associated with death (OR 22.4, 95% CI [1.77,283]; *p* = 0.016). Complications occurred only among DTC patients (8/307, compared with 0/362 HBC patients), so this estimate rests on few patients (Table 2).

Adding a restricted cubic spline of the date of notification to the primary model did not significantly improve fit (likelihood ratio test, *χ*^2^ = 0.60, 3 degrees of freedom, *p* = 0.895). The estimate for HBC with calendar time included was OR 0.35 (95% CI [0.08,1.57]; *p* = 0.170), with a wider interval than in the primary model (Table 2). The interaction between treatment modality and epidemic phase (early or late, split at the median notification date of 14 August 2023) could not be estimated: there were no deaths among the 92 HBC patients notified in the early phase, compared with 11 deaths among 245 DTC patients, and in the late phase there were 7 deaths among 270 HBC patients and 6 among 62 DTC patients.

Because LGA is constant within matched subclasses, adding it as a fixed covariate left the estimate unchanged by construction (OR 0.36, 95% CI [0.12,1.07]; *p* = 0.066). In an unconditional model with a random intercept for LGA, the odds ratio for HBC was 0.31 (95% CI [0.13,0.79]; *p* = 0.014).

### Long-term sequelae

Among 645 patients who survived, 14 (2.2%) reported long-term sequelae at the interview, a median of 421 days (range 251–624) after the date of notification: 8/290 (2.8%) in the DTC arm and 6/355 (1.7%) in the HBC arm. The sequelae reported were reinfection (6 patients), other complaints (4), neuritis (2), otitis media (2), malnutrition (1), and breathing problems (1); patients could report more than one.

In the primary model, which compares patients within matched subclasses, treatment modality was not clearly associated with sequelae (OR 0.63, 95% CI [0.18,2.22]; *p* = 0.471). Only 12 of the 141 subclasses with survivors contained both arms and both patients with and without sequelae, holding 7 DTC and 5 HBC events. In sensitivity analyses the odds ratio was 0.60 (95% CI [0.18,2.01]) for treatment alone, 0.64 (95% CI [0.22,1.80]) in a Firth penalised model, and 1.00 (95% CI [0.24,4.16]) among the 640 survivors without complications, and the design-based RR was 0.64 (95% CI [0.23,1.74]), with a risk difference of −1.0 percentage points (95% CI [−3.2,1.2]). Complications could not be estimated in the conditional model because all five survivors with complications were treated in a DTC; in the Firth model, complications were associated with higher odds of sequelae (OR 37.9, 95% CI [5.18,257]; *p* = 0.001), and two of the five had sequelae.

### Household transmission

The household rosters listed 5,043 members of the 669 index patients’ households. In the primary window of 2–14 days there were 136 secondary cases (SAR 2.7%, 95% CI [2.3,3.2]) in 95 of the 669 households (54/307 DTC and 41/362 HBC). The SAR was 2.2% (58/2,608; 95% CI [1.7,2.9]) among household members of HBC patients and 3.2% (78/2,435; 95% CI [2.5,4.0]) among those of DTC patients (Table 3).

**Table 3:** Household secondary attack rates by treatment modality and time window from index case symptom onset, Kano State diphtheria epidemic, Nigeria, 2023–2024.

Time window	HBC		DTC		Overall	
	Cases/N	SAR [95% CI]	Cases/N	SAR [95% CI]	Cases/N	SAR [95% CI]
2–14 days	58/2,608	2.2% [1.7,2.9]	78/2,435	3.2% [2.5,4.0]	136/5,043	2.7% [2.3,3.2]
2–30 days	77/2,608	3.0% [2.3,3.7]	90/2,435	3.7% [3.0,4.5]	167/5,043	3.3% [2.8,3.8]
Any timing	176/2,608	6.7% [5.8,7.8]	211/2,435	8.7% [7.6,9.9]	387/5,043	7.7% [7.0,8.4]

HBC, home-based care; DTC, diphtheria treatment centre; SAR, secondary attack rate, calculated as the number of household members developing symptoms within the specified window divided by the total number of household members listed. Index cases are excluded from both the numerator and the denominator. CI, confidence interval. CIs calculated using exact binomial methods.

In the logistic model for the primary window (4,728 household members, 136 cases), the odds of secondary infection were not clearly different between HBC and DTC (OR 0.71, 95% CI [0.44,1.16]; *p* = 0.174), and the RR was 0.72 (95% CI [0.45,1.15]; *p* = 0.173) (S3 Table). A model with a random intercept for household gave an OR of 0.65 (95% CI [0.27,1.55]; *p* = 0.335), with a latent-scale intraclass correlation of 0.86. The odds ratio was 0.81 (95% CI [0.51,1.29]; *p* = 0.378) in the window of 2–30 days (4,759 household members) and 0.78 (95% CI [0.57,1.07]; *p* = 0.123) with no restriction (4,979 household members).

### Acceptability

Acceptability scores were high in both arms. On a scale of 1–5, mean scores (standard deviation [SD]) in the DTC and HBC arms were 4.76 (0.50) and 4.66 (0.60) for acceptability, 4.78 (0.50) and 4.71 (0.53) for quality, 4.78 (0.59) and 4.78 (0.59) for perceived improvement, and 4.77 (0.43) and 4.72 (0.49) for the composite score. In the mixed models (669 patients, and 668 for quality and the composite), the estimated variance between subclasses was zero. HBC scored lower than DTC on acceptability by 0.113 points (95% CI [−0.198, −0.029]; *p* = 0.009). The differences were −0.070 points for quality (95% CI [−0.149,0.010]; *p* = 0.085), −0.007 points for perceived improvement (95% CI [−0.098,0.083]; *p* = 0.874), and −0.064 points for the composite score (95% CI [−0.135,0.007]; *p* = 0.078). A treatment delay of 4 days or more was associated with lower scores on acceptability (−0.122 points, 95% CI [−0.233, −0.011]; *p* = 0.032), quality (−0.112 points, 95% CI [−0.216, −0.008]; *p* = 0.035), perceived improvement (−0.119 points, 95% CI [−0.239,0.000]; *p* = 0.050), and the composite score (−0.118 points, 95% CI [−0.212, −0.024]; *p* = 0.014).

Cumulative-link models, with 95% CIs from a subclass bootstrap, gave odds ratios for a more favourable response with HBC compared with DTC of 0.61 (95% CI [0.42,0.87]) for acceptability, 0.66 (95% CI [0.44,1.00]) for quality, and 0.93 (95% CI [0.57,1.53]) for perceived improvement (S6 Table).

A treatment delay of 4 days or more was associated with lower odds of a more favourable response on all three dimensions (OR 0.58, 95% CI [0.40,0.86]; 0.58, 95% CI [0.40,0.82]; and 0.48, 95% CI [0.31,0.79], respectively).

### Sensitivity and robustness

In sensitivity analyses of the primary model, the odds ratio for HBC remained below 1. Excluding the eight patients with complications (661 patients, 21 deaths) gave an OR of 0.49 (95% CI [0.16,1.52]; *p* = 0.216), and a Firth penalised logistic regression gave an OR of 0.34 (95% CI [0.13,0.80]; *p* = 0.013). With the first card-based definition of vaccination (648 patients, 24 deaths) the OR was 0.27 (95% CI [0.08,0.89]; *p* = 0.031), and with the second (275 patients, 17 deaths) it was 0.47 (95% CI [0.13,1.64]; *p* = 0.234). The association did not differ by treatment delay (interaction OR 0.96 per day, 95% CI [0.54,1.71]; likelihood ratio test *p* = 0.896).

The composite of death or long-term sequelae occurred in 38 of 669 patients (24 deaths and 14 patients with sequelae among survivors): 13/362 (3.6%) in the HBC arm and 25/307 (8.1%) in the DTC arm. The OR for HBC was 0.40 (95% CI [0.18,0.89]; *p* = 0.025) in the primary model and 0.42 (95% CI [0.21,0.82]; *p* = 0.011) in a Firth penalised model. The *E*-value for the HBC estimate in the primary mortality model was 4.98, and 1.00 for the confidence limit closest to 1; the *E*-values were 5.70 (1.15 for the confidence limit) for vaccination and 1.91 (1.04) for treatment delay.

Stabilised IPTW, which estimates the association across all 669 patients, gave a marginal OR for HBC of 0.29 (95% CI [0.12,0.72]; *p* = 0.008) compared with 0.36 (95% CI [0.12,1.07]) in the primary model. The doubly robust OR was 0.30 (95% CI [0.12,0.75]; *p* = 0.010). The marginal RR was 0.31 (95% CI [0.13,0.73]; *p* = 0.008), and the marginal risk difference was −4.1 percentage points (95% CI [−7.1, −1.0]; *p* = 0.009), with weighted mortality of 5.9% in the DTC arm and 1.8% in the HBC arm. Propensity scores ranged from 0.27 to 0.79 in the DTC arm and from 0.25 to 0.79 in the HBC arm, with none below 0.05 or above 0.95, and 3 of 669 patients (0.4%) fell outside the region of common support (0.27 to 0.79) (S1 Fig). Effective sample sizes after trimming were 302 in the DTC arm and 357 in the HBC arm (S4 Table). After weighting, standardised mean differences were 0.004 for age, 0.008 for vaccination status, and 0.015 for household size, compared with 0.032, 0.088, and 0.191 before weighting. The marginal OR was 0.29 (95% CI [0.12,0.72]; *p* = 0.007) without trimming and 0.30 (95% CI [0.12,0.74]; *p* = 0.009) with trimming at the 5th and 95th percentiles.

Weighting the IPTW estimates by the inverse probability of being interviewed gave a marginal OR for HBC of 0.32 (95% CI [0.13,0.80]; *p* = 0.014) and a doubly robust OR of 0.32 (95% CI [0.13,0.81]; *p* = 0.015). The weights ranged from 0.82 to 2.25, with a mean of 1.00 for the originally sampled patients and 1.06 for replacements. Among the interviewed replacements, 5/57 (8.8%) in the DTC arm and 4/121 (3.3%) in the HBC arm died, compared with 12/250 (4.8%) and 3/241 (1.2%) among the originally sampled patients who were interviewed. In the 491 originally sampled patients who were interviewed (15 deaths), the primary model gave an OR for HBC of 0.10 (95% CI [0.01,0.80]; *p* = 0.030); 12 of the 15 deaths were in 9 subclasses that contained both arms and a difference in outcome, and only one of these deaths was in the HBC arm. In this subset the Firth OR was 0.27 (95% CI [0.07,0.84]; *p* = 0.023) and the IPTW OR was 0.21 (95% CI [0.06,0.77]).

In an unconditional logistic regression with the same covariates as the primary model, variance inflation factors were at most 1.20 and the c-statistic was 0.83. In the highest quintile of predicted risk, predicted mortality was 12.0% and observed mortality was 12.8% (S2 Table). The within-subclass concordance of the primary conditional model was 0.77 (standard error [SE] 0.07). The largest absolute dfbeta for the HBC coefficient was 0.22 on the log-odds scale.

## Discussion

During a large diphtheria epidemic that overwhelmed hospital capacity in Kano State, home-based care for patients with mild disease was not associated with higher mortality than facility-based treatment. We observed no increase in sequelae or household transmission associated with HBC, and acceptability was high in both arms. Mortality was associated with treatment delay and vaccination status, while we found no clear evidence of an independent association with care setting.

Guidance for diphtheria recommends hospital admission, DAT, and monitoring for cardiac and neurological complications [1,2], a standard that large epidemics in resource-limited settings can exceed. In a large measles epidemic in the Democratic Republic of the Congo, a comprehensive case-management approach was associated with lower case fatality, and children who lived farther from the hospital had a higher risk of death [11]. Decentralised models of care have also been evaluated during Ebola and cholera responses [12–14]. Early antibiotics halt toxin production in diphtheria [2], which makes outpatient management of mild disease clinically plausible. In both arms of this study, patients with mild disease were treated with antibiotics and did not routinely receive DAT, so the arms differed in where care was delivered. To our knowledge, no published study has compared home-based or community-based clinical management of diphtheria with facility-based care (PubMed search, October 2026).

The OR for death with HBC in the primary model, which rests on subclasses that contained both arms, was 0.36 (95% CI [0.12,1.07]; *p* = 0.066). The population-averaged estimates include the 72 patients in single-arm subclasses and had intervals that excluded 1: the RR was 0.36, the marginal OR from IPTW was 0.29, and the doubly robust OR was 0.30. The gap between 0.36 and 0.29 reflects the different patients and adjustment in the two analyses. The conditional model compares patients within subclasses defined by ward and excludes single-arm subclasses, whereas IPTW uses all 669 patients and balances LGA rather than ward. Adding age, vaccination status, and treatment delay changed the IPTW estimate only from 0.29 to 0.30. Non-collapsibility would place the conditional OR further from 1 than the marginal OR, so it does not explain the gap. The composite of death or long-term sequelae pointed in the same direction (OR 0.40). Even at the protocol’s target of 496 patients per group, the study would have had about 55% power, so an interval that includes 1 in the primary model is not evidence of no difference. All patients with complications or comorbidities were treated in DTCs, so residual confounding by severity could account for part of the lower mortality with HBC, and these results do not show that HBC reduced mortality.

Each additional day between symptom onset and the start of treatment was associated with 29% higher odds of death. In the companion survey, the risk of death was higher among people who waited four days or more before seeking care, although the estimate was imprecise (RR 24.3, 95% CI [2.97,199], based on 8 deaths) [7]. DAT neutralises circulating toxin but cannot reverse damage already done [2], which provides a plausible mechanism for the association with delay. Possible contributors to delay in this setting include shortages of DAT, which have been reported across the regional resurgence [15,16], delayed access to antibiotics [15], distance to treatment sites, transport costs, and limited awareness of diphtheria. Median delay was 2 days in both arms, so this study cannot show whether HBC shortens delay.

Vaccination was associated with lower odds of death (OR 0.32), and the estimate was similar with the first card-based definition (OR 0.23). With the second definition, which excluded unverified reports and left 275 patients, the estimate was imprecise (OR 0.44, 95% CI [0.05,4.06]). Vaccination status was reported at interview. Some doses may have been given after notification, and patients who died early could not receive them, which could overstate the association.

Long-term sequelae were uncommon (14 of 645 survivors) and did not differ clearly between arms. Only 12 subclasses were informative for this estimate, so the analysis cannot show whether HBC changes the risk of sequelae in either direction. Sequelae were reported at a single time point, six of the 14 patients reported reinfection, and only survivors were analysed. The SAR was lower in the HBC arm in all three windows. No OR for household transmission excluded 1, so HBC was not associated with higher household transmission, but a difference in either direction cannot be excluded. Secondary cases were reported by respondents at the interview, and the SAR intervals treat household members as independent.

Acceptability was high in both arms, which limits the room for differences. HBC scored 0.11 points lower than DTC care on the acceptability item on a 5-point scale, with no clear difference in quality, perceived improvement, or the composite score. Longer treatment delay was associated with lower scores on all four measures. Our data do not explain the lower acceptability score with HBC.

The main limitation is residual confounding by disease severity. Allocation followed clinical triage, and all recorded complications and comorbidities occurred among DTC patients, which suggests that patients with more complex presentations were more often admitted. Mild disease was defined clinically, and severity within that group was not measured in more detail. Matching and weighting cannot adjust for unmeasured differences such as pseudomembrane extent. An unmeasured confounder associated with both DTC admission and death by RRs of about 5 or more could explain away the point estimate for HBC (*E*-value 4.98, assuming death is rare enough for the OR to approximate the RR), and associations of that size between severity and both admission and death are plausible. The *E*-value for the interval limit was 1.00, because the interval includes 1. Complications were recorded by clinicians after allocation, may lie on the pathway to death, and may have been ascertained differently between arms, so the estimates that adjust for or exclude them are sensitivity analyses and not severity-free estimates. Cases were defined clinically, without laboratory confirmation.

With 24 deaths and four covariates, the primary model gives imprecise estimates, as the wide intervals show. The Firth analysis gave a more precise estimate, but it does not condition on matched subclasses and includes patients in single-arm subclasses, so its precision does not come from small-sample correction alone. HBC was introduced in July 2023, so treatment arm and calendar time are closely related. Adding calendar time left the HBC estimate essentially unchanged and widened its interval, and the test for calendar time had little power with 24 deaths and 3 degrees of freedom, so it cannot rule out confounding by time. The LGA random intercept is poorly estimated with only four LGAs, and matching on ward does not account for correlation of outcomes within LGAs. We did not adjust for multiple comparisons across outcomes and sensitivity analyses.

Fewer than half of the originally sampled patients were interviewed (491 of 986). They differed from those who were not by sex and vaccination status but not by treatment arm. Replacements made up more than a quarter of the interviewed patients and a third of the HBC arm, were assigned to strata and not to named patients, and sometimes crossed arms. The selection-weighted estimate was close to the main IPTW estimate, but it assumes that the probability of interview for replacements resembles that of original patients with the same registry characteristics, and this cannot be verified. Replacements died more often than originally sampled patients in both arms, and we cannot say whether this reflects selection or chance. Among originally sampled patients alone the OR was much lower, but this estimate is unstable and not confirmatory.

Deaths, sequelae, vaccination status, treatment delay, and acceptability were reported by patients or caregivers at an interview typically more than a year after notification, so recall error is possible, and acceptability ratings may have been influenced by the interview setting. Deaths were all-cause and ascertained up to the interview, we did not analyse time to death, and DTC patients, who were notified earlier, were followed for longer. Eligibility required a telephone number, so households without one were not represented. The study took place in four LGAs of Kano State within an MSF-supported programme with structured triage, follow-up visits, and a referral pathway, and the findings may not apply where these are absent.

HBC could be considered during diphtheria epidemics for patients with mild disease, provided that triage reliably distinguishes mild from moderate and severe disease, antibiotic treatment is initiated upon presentation, and follow-up and a functioning referral pathway are in place. Under these conditions, it could free hospital beds and DAT for patients with more severe disease. Diphtheria continues to spread across West and Central Africa [15,17], where hospital capacity may again be limited.

These findings suggest that HBC may provide an additional model of care for carefully selected patients when hospital capacity is constrained, provided that clinical triage, follow-up, and referral systems are in place. Confirmation of these findings through a prospective, ideally randomised, study across additional settings and larger sample sizes would strengthen confidence in HBC as a routine component of diphtheria epidemic response, and would allow the residual allocation bias inherent to this observational design to be directly addressed.

## Supporting information

S1 Checklist STROBE Statement checklist of items to include in reports of cohort studies. Source: von Elm E, and colleagues. PLoS Med. 2007;4(10):e296 (https://doi.org/10.1371/journal.pmed.0040296; https://www.strobe-statement.org/), licensed CC BY 4.0.
(DOCX)

S2 Checklist PLOS Inclusivity in Global Research questionnaire. (DOCX)

S1 File Study protocol, version 2024-07-08, approved by the MSF Ethics Review Board on 9 July 2024 (reference 2437). (DOCX)

S2 File Departures from the study protocol (version 2024-07-08) in sampling, matching, replacement of patients, outcomes, and analysis. (DOCX)

S3 File Interview questionnaire administered to patients or their caregivers with KoboCollect. (DOCX)

S1 Text Software, R packages, and model specifications used in the analyses. (DOCX)

S1 Table Complete-case sample size and number of events included in each mortality, long-term sequelae, household transmission, and acceptability model reported in the manuscript. (DOCX)

S2 Table Diagnostics for the primary mortality model (Model A): variance inflation factors, discrimination (c-statistic), and calibration by predicted-risk quintile from an unconditional logistic regression with the same covariates, and within-subclass concordance of the conditional model, and case-level influence (dfbeta) from an Efron-method refit of the conditional model. (DOCX)

S3 Table Risk ratios alongside adjusted odds ratios for covariates in the multivariable household secondary attack rate model. (DOCX)

S4 Table Inverse probability of treatment weighting (IPTW) diagnostics: propensity score range and common-support overlap, covariate balance (standardised mean differences) before and after weighting, effective sample size before and after trimming, and trimming sensitivity analysis. (DOCX)

S5 Table Standardised mean differences between treatment arms among the 669 interviewed patients, including replacements, and comparison of interviewed and not interviewed patients among the 986 originally sampled on baseline registry variables. (DOCX)

S6 Table Cumulative-link (ordinal) sensitivity models for each patient-reported acceptability Likert dimension (acceptability, quality, perceived improvement). (DOCX)

S1 Fig Propensity score distributions by treatment arm in the inverse probability of treatment weighting analysis. The region of common support was 0.27 to 0.79.
(TIF)

## Abbreviations

AI: artificial intelligence
CFR: case fatality risk
CI: confidence interval
DAT: diphtheria antitoxin
DTC: diphtheria treatment centre
HBC: home-based care
IPTW: inverse probability of treatment weighting
IQR: interquartile range
LGA: Local Government Area
MSF: Médecins Sans Frontières
OR: odds ratio
RR: risk ratio
SAR: secondary attack rate
SD: standard deviation
SE: standard error
