# Supplementary material for: Outcomes of home-based versus facility-based care for mild diphtheria during a large epidemic in Kano State, Nigeria: A retrospective matched cohort study
Source: PLoS Med. 2026 Oct 26;23(10):e1005097. doi: 10.1371/journal.pmed.1005097 (PMC13722324; doi:10.1371/journal.pmed.1005097)
Supplement: Supplementary file 1 [file pmed.1005097.s001.docx]

**S1 Checklist. STROBE statement: checklist of items that should be included in reports of cohort studies.**

*Applied to: Outcomes of home-based versus facility-based care for mild diphtheria during a large epidemic in Kano State, Nigeria: a retrospective matched cohort study (Polonsky et al.).*

| **Section** | **Item No** | **Recommendation** | **Reported in manuscript (section, paragraph)** |
| --- | --- | --- | --- |
| Title and abstract | 1(a) | Indicate the study's design with a commonly used term in the title or the abstract | *Title (“...a retrospective matched cohort study”); Abstract, Methods paragraph* |
|  | 1(b) | Provide in the abstract an informative and balanced summary of what was done and what was found | *Abstract (Background, Methods and Findings, Conclusions paragraphs)* |
| **Introduction** | | | |
| Background/rationale | 2 | Explain the scientific background and rationale for the investigation being reported | *Introduction, ¶1 to ¶4* |
| Objectives | 3 | State specific objectives, including any prespecified hypotheses | *Introduction, final paragraph* |
| **Methods** | | | |
| Study design | 4 | Present key elements of study design early in the paper | *Methods: Study design and setting, ¶1* |
| Setting | 5 | Describe the setting, locations, and relevant dates, including periods of recruitment, exposure, follow-up, and data collection | *Methods: Study design and setting, ¶1; Participants and matching, ¶1 (patients notified January 2023 to May 2024; interviews September to October 2024)* |
| Participants | 6(a) | Give the eligibility criteria, and the sources and methods of selection of participants. Describe methods of follow-up | *Methods: Participants and matching, ¶1 to ¶3* |
|  | 6(b) | For matched studies, give matching criteria and number of exposed and unexposed | *Methods: Participants and matching, ¶1 (sex, age group, vaccination status, and ward of residence; 496 DTC and 490 HBC originally sampled); Results: Participants, ¶1 (669 interviewed: 307 DTC and 362 HBC)* |
| Variables | 7 | Clearly define all outcomes, exposures, predictors, potential confounders, and effect modifiers. Give diagnostic criteria, if applicable | *Methods: Definitions and Outcomes, full sections* |
| Data sources/measurement | 8* | For each variable of interest, give sources of data and details of methods of assessment. Describe comparability of assessment methods if there is more than one group | *Methods: Data collection, full section* |
| Bias | 9 | Describe any efforts to address potential sources of bias | *Methods: Participants and matching, ¶1 to ¶3 (exact matching, replacement protocol, allocation); Statistical analysis, ¶6 to ¶7 (calendar time, LGA); Sensitivity and robustness analyses, ¶2 to ¶3 (IPTW, selection weights); Discussion, ¶8 to ¶10 (residual confounding, imprecision, selection)* |
| Study size | 10 | Explain how the study size was arrived at | *Methods: Sample size, full section* |
| Quantitative variables | 11 | Explain how quantitative variables were handled in the analyses. If applicable, describe which groupings were chosen and why | *Methods: Statistical analysis, ¶4 (age and treatment delay modelled continuously, with a spline check for delay); Statistical analysis, ¶9 to ¶10 (treatment delay of 4 days or more in the household transmission and acceptability models)* |
| Statistical methods | 12(a) | Describe all statistical methods, including those used to control for confounding | *Methods: Statistical analysis, full section* |
|  | 12(b) | Describe any methods used to examine subgroups and interactions | *Methods: Statistical analysis, ¶6 (epidemic-phase interaction); Sensitivity and robustness analyses, ¶1 (treatment-by-delay interaction); Results: Mortality, ¶4; Sensitivity and robustness, ¶1* |
|  | 12(c) | Explain how missing data were addressed | *Methods: Sensitivity and robustness analyses, ¶3 (multiple imputation not used); S1 Table (patients included in each model); Discussion, ¶10* |
|  | 12(d) | If applicable, explain how loss to follow-up was addressed | *Methods: Participants and matching, ¶2 (replacement protocol); Sensitivity and robustness analyses, ¶3 (selection weights); Discussion, ¶10* |
|  | 12(e) | Describe any sensitivity analyses | *Methods: Sensitivity and robustness analyses, full section* |
| **Results** | | | |
| Participants | 13(a) | Report numbers of individuals at each stage of study | *Results: Participants, ¶1; Figure 1* |
|  | 13(b) | Give reasons for non-participation at each stage | *Methods: Participants and matching, ¶2; Results: Participants, ¶1; Figure 1* |
|  | 13(c) | Consider use of a flow diagram | *Figure 1* |
| Descriptive data | 14(a) | Give characteristics of study participants and information on exposures and potential confounders | *Results: Participants, ¶4 to ¶5; Table 1* |
|  | 14(b) | Indicate number of participants with missing data for each variable of interest | *S1 Table (patients included in each model); Results: Mortality, ¶2 (patients with card-based vaccination data)* |
|  | 14(c) | Summarise follow-up time | *Results: Long-term sequelae, ¶1 (median 421 days, range 251 to 624 days)* |
| Outcome data | 15* | Report numbers of outcome events or summary measures over time | *Results: Mortality, ¶1; Household transmission, ¶1; Long-term sequelae, ¶1; Acceptability, ¶1* |
| Main results | 16(a) | Give unadjusted and confounder-adjusted estimates and their precision. Make clear which confounders were adjusted for and why | *Results: Mortality, full section; Table 2; Household transmission; Table 3* |
|  | 16(b) | Report category boundaries when continuous variables were categorized | *Methods: Participants and matching, ¶1 (age groups); Statistical analysis, ¶9 to ¶10 (treatment delay of 4 days or more)* |
|  | 16(c) | If relevant, consider translating estimates of relative risk into absolute risk | *Results: Mortality, ¶1 (case fatality risk and RR); Sensitivity and robustness, ¶3 (risk difference); Table 1* |
| Other analyses | 17 | Report other analyses done | *Results: Mortality, ¶3 to ¶5; Long-term sequelae; Acceptability; Sensitivity and robustness* |
| **Discussion** | | | |
| Key results | 18 | Summarise key results with reference to study objectives | *Discussion, ¶1* |
| Limitations | 19 | Discuss limitations of the study, taking into account sources of potential bias or imprecision | *Discussion, ¶8 to ¶11 (residual confounding, imprecision, selection, recall, and generalisability)* |
| Interpretation | 20 | Give a cautious overall interpretation of results | *Discussion, ¶3 to ¶7 and ¶12 to ¶13* |
| Generalisability | 21 | Discuss the generalisability (external validity) of the study results | *Discussion, ¶11 (setting and programme conditions) and ¶12 to ¶13* |
| **Other information** | | | |
| Funding | 22 | Give the source of funding and the role of the funders | *Other information: Funding: “The author(s) received no specific funding for this work.”* |

**Give information separately for exposed and unexposed groups (i.e. HBC and DTC), as done throughout Results and Tables 1 to 3.*

*Source checklist: von Elm E, Altman DG, Egger M, Pocock SJ, Gøtzsche PC, Vandenbroucke JP; STROBE Initiative. The Strengthening the Reporting of Observational Studies in Epidemiology (STROBE) statement: guidelines for reporting observational studies. PLoS Med. 2007;4(10):e296.*
