# Supplementary material for: Outcomes of home-based versus facility-based care for mild diphtheria during a large epidemic in Kano State, Nigeria: A retrospective matched cohort study
Source: PLoS Med. 2026 Oct 26;23(10):e1005097. doi: 10.1371/journal.pmed.1005097 (PMC13722324; doi:10.1371/journal.pmed.1005097)
Supplement: Supplementary file 4 [file pmed.1005097.s004.docx]

**S2 File. Departures from the study protocol (version 2024-07-08) in sampling, matching, replacement of patients, outcomes, and analysis.**

| **Item** | **Protocol** | **As conducted** | **Reason and consequence** |
| --- | --- | --- | --- |
| Primary endpoint and sample size | The cohort objective was to evaluate the performance of home-based care in terms of treatment outcome and household secondary attack rates. The sample size was calculated for a comparison of the proportion with death or long-term sequelae between DTC and HBC care: expected case fatality risk 2·5% in DTC and 5% in HBC, two-sided alpha 0·05, 80% power, 10% non-response, 496 patients per group (992 in total). | All-cause mortality was the principal primary outcome; long-term sequelae and household secondary attack rate were the other primary outcomes. The composite of death or sequelae was analysed as a supplementary outcome (post hoc). We interviewed 669 patients (307 DTC, 362 HBC). | A repeat of the calculation gives about 905 patients per group, and about 55% power at 496 per group. Mortality had 24 events, so estimates are imprecise. |
| Statistical analysis | Descriptive statistics (proportions with 95% confidence intervals; medians with interquartile ranges), with Pearson chi-squared, Student t, or Wilcoxon rank-sum tests where appropriate. No models were specified for the comparison of HBC with DTC care. | Conditional logistic regression stratified by matched subclass (primary), with sensitivity analyses added during analysis and in response to peer review, identified as post hoc in the Methods. | The analysis section of the protocol did not cover the matched design that was implemented. |
| Study sites | Patients recorded as mild in the MSF database within the catchment areas of the Infectious Diseases Hospital (IDH) and the Murtala Muhammed Specialist Hospital (MMSH). | Three study sites: IDH and MMSH, and the Waziri Shehu Gidado General Hospital (WSGGH), which contributed HBC patients only. | WSGGH was an MSF-supported site that treated mild patients through HBC only. Excluding it would have removed 720 of the 2,494 HBC patients (28·9%) from the sampling frame. It contributed no DTC patients, so DTC patients came from IDH and MMSH. Of the 669 interviewed patients, 88 (all HBC) were from WSGGH. |
| Eligibility | Recorded as a mild patient in the MSF database in the catchment areas of IDH and MMSH, with written informed consent. No criteria on readmission or telephone number. | Mild disease at initial assessment, notified between 1 January 2023 and 31 May 2024, resident in one of the four LGAs, not readmitted, and with a unique 10-digit telephone number, so that the interview could be arranged. | Patients in households without a telephone are not represented. |
| Matching variables | Matching on sex, age group (five-year bands), and ward of residence (LGA if ward was not available). Vaccination status was not a matching variable. | Exact matching on sex, four age groups (0 to 5, 6 to 10, 11 to 15, and 16 years or more), ward of residence, and registry vaccination status. | Four age groups kept strata populated, because five-year bands left many strata without a patient of the other arm. Vaccination status was added because it is strongly associated with death and could differ between arms. Ward of residence was available in the registry for all patients. |
| Sampling | 496 patients classified as mild selected by simple random sampling from the list of patients treated in DTCs, and an equal number of HBC patients selected at random using sex, age group (five-year bands), and ward of residence (LGA if the ward was not available) as matching variables. | We selected 496 DTC patients from strata that contained both arms, allocating the number per LGA in proportion to eligible patients and choosing at random with probability proportional to the number of DTC and HBC pairs in the stratum, then drew as many HBC patients per stratum. Four strata had fewer eligible HBC than sampled DTC patients and all were sampled. The sample was 986 patients (496 DTC, 490 HBC), not 992. | Not a simple random sample and not fixed 1:1 pairs: exact strata gave 143 variable-size subclasses among the interviewed patients. |
| Contact attempts and exclusion | A patient not available after two visit attempts (morning and afternoon), or unwilling to respond, was excluded. | Field teams made two contact attempts by telephone, then arranged a home visit. Patients not reached after two attempts, who declined, or who were not interviewed at the visit were replaced. | Attempts were recorded only on a planning form filled after the second attempt; records exist only from 30 September 2024, and only for originally sampled patients. |
| Replacement of patients | The protocol made no provision for replacement. | Replacement from unsampled patients of the same stratum and arm; then from another stratum with the same age group, sex, vaccination status, and LGA (a different ward) and the same arm; then from such a stratum of either arm. Replacements were assigned to strata, not to named patients. 178 replacements were interviewed (57 DTC, 121 HBC). | Replacements made up more than a quarter of the interviewed patients and some crossed arms. Selection-weighted and originally-sampled-only analyses are reported in the Results (post hoc). |
| Timing of interviews | Survey period July to August 2024 (one month). | Interviews took place between 18 September and 21 October 2024. | Long-term outcomes were assessed a median of 421 days after notification. |
| Clinical records | Clinical data previously recorded in MSF databases were to be analysed for further detail and verification of clinical presentation and treatment history. | Clinical variables came from the MSF registry line list only; no patient charts were reviewed. | The MSF registry line list held the clinical variables needed (complications, admission location, and date of notification) for patients in both arms. Charts for patients treated at home were not available in a comparable form, and review of charts at three sites was not feasible, so the registry was the only uniform source. Clinical detail is limited to registry variables. |
| Acceptability | The questionnaire (Appendix 8) included acceptability questions; acceptability was not a stated objective or outcome. | Patient-reported acceptability was a secondary outcome, analysed with linear mixed models and, as a sensitivity analysis, cumulative-link models (post hoc). | The questionnaire collected acceptability data because patient experience of home care was of programme interest. We analysed them as a secondary outcome, labelled as such, to report all the outcomes collected; cumulative-link models were added in response to peer review (post hoc). |
| Analyses added after the protocol (post hoc) | Not specified. | Inverse probability of treatment weighting (marginal and doubly robust), selection-weighted analysis, analysis of originally sampled patients only, Firth penalised models, calendar-time and epidemic-phase models, LGA models, treatment by delay interaction, card-based vaccination definitions, composite outcome, design-based risk ratios, cumulative-link acceptability models, and E-values. | Added in response to peer review or developed during analysis; those added in response to peer review are labelled post hoc in the Methods. |
