# Supplementary material for: Outcomes of home-based versus facility-based care for mild diphtheria during a large epidemic in Kano State, Nigeria: A retrospective matched cohort study
Source: PLoS Med. 2026 Oct 26;23(10):e1005097. doi: 10.1371/journal.pmed.1005097 (PMC13722324; doi:10.1371/journal.pmed.1005097)
Supplement: Supplementary file 5 [file pmed.1005097.s005.docx]

## Retrospective cohort study questionnaire

| **CORE QUESTIONS** |  |  |
| --- | --- | --- |
| **Variable** | **Values** | **Comments** |
| **Information Note** | **Diphtheria Cohort Study, Kano State, Nigeria, 2024** |  |
| Date of interview | Select date from calendar function | Restricted to survey dates |
| Team ID | Team 1 : Team 10 | Team ID number |
| Patient ID | Numeric |  |
| Written informed consent given by the respondent | Y = Yes N = No |  |
| Address of household | Free text | For comparison with records to assess biases in residential location recording |
| GPS coordinates of household | Lon & Lat (and precision of point) |  |
| How many people are currently living in the household? | Numeric |  |
| *Diphtheria vaccination status* |  |  |
| Have you ever received diphtheria vaccination? | 0 = No  1 = Yes |  |
| If yes: Number of doses according to vaccination card | 0 = No card  1 = Yes (1 dose)  2 = Yes (2 doses)  3 = Yes (3 or more doses) |  |
| If card:  Date of vaccination (most recent dose) | (dd/mm/yyyy) |  |
| If no card:  Approximate vaccination date | (MM YYYY) |  |
| Is this individual aged 15 or under? | 0 = No  1 = Yes | Options available will include known periods of campaigns |
| If yes, did they receive the vaccine provided during the campaign? | 0 = No  1 = Yes (card observed)  2 = Yes (card not observed) | Specify months of campaign in question |
| If no, reason why not vaccinated | 1 = Did not think it was needed  2 = Did not know where to get vaccination  3 = Did not know where to get good/reliable information  4 = Heard or read negative media  5 = Did not think the vaccine was effective  6 = Did not think the vaccine was safe/concerned about side effects  7 = Someone else told me that the vaccine was not safe  8 = Had a bad experience with previous vaccinator/health clinic  9 = Had a bad experience or reaction with previous vaccination  10 = Fear of needles  11 = Not possible to leave other work  12 = Religious reasons  13 = Refusal of male head of household  14 = Belief it can lead to infertility  15 = Already vaccinated against diphtheria  16 = Other beliefs/traditional medicine  17 = Other (please specify): |  |
| Was your household visited by an MSF health promotion worker who provided information about diphtheria and vaccination? | 0 = No  1 = Yes  99 = Do not recall |  |
| If yes, did this change how likely you would be to get vaccinations for yourself and your family? | 0 = No impact  1 = More likely  2 = Less likely |  |
| *Diphtheria history* |  |  |
| What were your symptoms? | 1 = Whitish/greyish layer of the tongue  2 = Swollen neck  3 = Fever  4 = Sore throat  5 = Difficulty swallowing  6 = Difficulty breathing  7 = Drooling saliva  8 = Nasal regurgitation  9 = Nasal blood  10 = Malaise/Lethargy  11 = Loss of appetite  12 = Hoarseness  99 = None of the above | Check all that apply  Will need community language for these |
| What was the outcome of illness? | 1 = Recovered  2 = Still unwell  3 = Died |  |
| If still unwell, what are the long-term health problems? | 1 = Myocarditis/heart failure  2 = Neuritis  3 = Kidney failure  3 = Otitis media  4 = Problems breathing  5 = Malnutrition  6 = Other (specify) | Check all that apply  Will need community language for these |
| Where was the first place you sought healthcare? | 1 = Health centre (*specify*)  2 = Diphtheria Treatment Centre (*specify*)  3 = Hospital *(specify)*  4 = Traditional healer  5 = Pharmacy  6 = Other place *(specify)*  99 = DNK |  |
| If not at a health facility, what was the main reason for not seeking care in a health structure? | 1 = No money/consultation too expensive  2 = Too sick to seek care  3 = Not sick enough to seek care 4 = Health facility too far away 5 = Went to traditional healer  6 = Did not have time/too busy to go 7 = No confidence in health system  8 = Security problem 9 = Care was refused at facility  10 = Other reason *(specify)*  99 = DNK |  |
| What was the delay between disease onset and treatment beginning with MSF? | Numeric (days) |  |
| Where were you treated by MSF? | 1 = At health facility  2 = At home |  |
| What treatment did you receive? | 0 = None  1 = Antibiotic only  2 = Antitoxin only  3 = Antibiotic and antitoxin  4 = Other/traditional treatment  99 = DNK |  |
| Did your $home-based/$hospital-based treatment lead to an improvement in the condition? | 1 = Strongly disagree  2 = Disagree  3 = No opinion  4 = Agree  5 = Strongly agree  99 = DNK |  |
| If quite/strongly negative, please explain | Free text |  |
| How acceptable was the $home-based/$hospital-based treatment to you? | 1 = Completely unacceptable  2 = Unacceptable  3 = No opinion  4 = Acceptable  5 = Completely acceptable  99 = DNK |  |
| If quite/strongly negative, please explain | Free text |  |
| How do you feel about the quality of treatment you received? | 1 = Strongly positive  2 = Quite positive  3 = Neutral  4 = Quite negative (specify)  5 = Strongly negative (specify) |  |
| If quite/strongly negative, please explain | Free text |  |
| If at home, how do you feel about being treated at home rather than in a health facility? | 1 = Strongly positive  2 = Quite positive  3 = Neutral  4 = Quite negative (specify)  5 = Strongly negative (specify) |  |
| If quite/strongly negative, please explain | Free text |  |
| Did anyone else in your household show signs of diphtheria prior to you developing symptoms? | 0 = No 1 = Yes  99 = DNK |  |
| If yes, what was the delay between their disease beginning and yours beginning? | Numeric (days/weeks) |  |
| Did anyone else in your household show signs of diphtheria after you developed symptoms? | 0 = No 1 = Yes  99 = DNK |  |
| If yes, how many people? | Numeric |  |
| If yes, what was the delay between the beginning of your disease and the next person to fall ill? | Numeric (days/weeks) |  |
| *For other household members* |  |  |
| Household member order | Numeric |  |
| Sex | M = Male  F = Female |  |
| Age | Numeric (months/years) |  |
| Did this person ever receive diphtheria vaccination? | 0 = No  1 = Yes |  |
| If yes: Number of doses according to vaccination card | 0 = No card  1 = Yes (1 dose)  2 = Yes (2 doses)  3 = Yes (3 or more doses) |  |
| If card:  Date of vaccination (most recent dose) | (dd/mm/yyyy) |  |
| If no card:  Approximate vaccination date | (MM YYYY) |  |
| Did this individual have suspected diphtheria during the recall period | 0 = No 1 = Yes  99 = DNK | Clear case definitions necessary |
| If yes, did they develop symptoms before or after the subject of this investigation? | 1 = Before  2 = After  99 = Do not recall |  |
| If yes, what was the outcome? | 1 = Recovered  2 = Still unwell (complications)  3 = Died |  |
| If yes, approximate date of disease onset? | (mm/yy) | Event calendar to help |
| If yes, were they vaccinated prior to disease onset? | 0 = No  1 = Yes (any doses)  99 = DNK | Asking again for extra certainty |
| If yes, did they provide care to another diphtheria patient within the household prior to developing symptoms? | 0 = No 1 = Yes  99 = DNK |  |
| If yes, what were their symptoms? | 1 = Whitish/greyish layer of the tongue  2 = Swollen neck  3 = Fever  4 = Sore throat  5 = Difficulty swallowing  6 = Difficulty breathing  7 = Drooling saliva  8 = Nasal regurgitation  9 = Nasal or other bleeding  10 = Malaise/Lethargy  11 = Loss of appetite  12 = Hoarseness  99 = None of the above | Check all that apply  Will need community language for these |
| If yes, was there a diagnosis by a health professional? | 0 = No  1 = Yes  99 = DNK |  |
| If yes, did they seek healthcare? | 0 = No 1 = Yes  99 = DNK |  |
| If yes, what treatment did they receive? | 0 = None  1 = Antibiotic only  2 = Antitoxin only  3 = Antibiotic and antitoxin  4 = Other/traditional treatment  99 = DNK |  |
| If yes, place health care sought? | 1 = Home  2 = Health centre  3 = Diphtheria Treatment Centre (*specify*)  4 = Private hospital *(specify)*  5 = Public hospital (*specify*)  6 = Traditional healer  7 = Patient Medicine Vendor/Pharmacy  8 = Other place *(specify)*  99 = DNK | Select all that apply, in order of visit |
| If yes, after seeking healthcare, where were they finally treated? | 1 = Health facility (specify)  2 = Home  99 = DNK | This is to distinguish between those receiving DTC-based and home-based care |
| If yes, who treated them? | 1 = MSF  2 = Other NGO (e.g. Alima/Save The Children) 3 = Government health facilities not supported by NGOs  99 = DNK |  |
| If yes, delay between disease onset and treatment beginning? | Numeric (days) | This refers to diphtheria treatment by one of the providers above (NGO/govt) |
| If no, main reason for not seeking care in a health structure? | 1 = No money/consultation too expensive  2 = Too sick to seek care  3 = Not sick enough to seek care 4 = Health facility too far away 5 = Went to traditional healer  6 = Did not have time/too busy to go 7 = No confidence in health system  8 = Security problem 9 = Care was refused at facility  10 = Refusal of male head of household 11 = Died suddenly before visit could be arranged  12 = Other reason *(specify)* 99 = DNK |  |

Note on coding for the analysis: the quality item is coded 1 = strongly positive to 5 = strongly negative in the questionnaire. In the analysis it was reversed (strongly positive = 5, strongly negative = 1), so that higher scores indicate a more favourable response, as for the other two acceptability items.
