# Supplementary material for: Outcomes of home-based versus facility-based care for mild diphtheria during a large epidemic in Kano State, Nigeria: A retrospective matched cohort study
Source: PLoS Med. 2026 Oct 26;23(10):e1005097. doi: 10.1371/journal.pmed.1005097 (PMC13722324; doi:10.1371/journal.pmed.1005097)
Supplement: Supplementary file 6 [file pmed.1005097.s006.docx]

**S1 Text. Software, R packages, and model specifications used in the analyses.**

All analyses were run in R version 4.5.2 (2025-10-31). The code that prepares the de-identified analysis files (hbc_prep.R) and reproduces the results (hbc_results.R) is publicly available (see Data sharing); the sampling and matching code ran on the registry data and is not included. All tests were two-sided, confidence intervals (CIs) are 95% Wald intervals unless stated, and no adjustment was made for multiple comparisons. In the formulas below, treatment is HBC versus DTC, age is in years, delay is treatment delay in days, vaccinated is vaccination status at interview, and subclass is the matched stratum (exact match on age group, sex, ward of residence, and registry vaccination status).

**Software and packages**

| **Package and version** | **Use** |
| --- | --- |
| survival 3.8.9 | Conditional logistic regression (clogit, exact partial likelihood), concordance |
| survey 4.5, srvyr 1.3.1 | Design-based risk ratios, risk differences, and case fatality risks, with subclass as stratum |
| lme4 2.0.6, lmerTest 3.2.1 | Linear and logistic mixed-effects models; p-values for linear mixed models |
| ordinal 2026.7.26 | Cumulative-link models (clm, clmm) |
| logistf 1.26.1 | Firth penalised logistic regression |
| sandwich 3.1.3, lmtest 0.9.40 | Heteroscedasticity-robust (HC1) and cluster-robust standard errors |
| rms 8.1.1 | Restricted cubic splines and likelihood ratio tests |
| car 3.1.5 | Variance inflation factors |
| EValue 4.1.4 | E-values |
| MatchIt 4.7.2 | Exact matching of the sampling frame |
| epiR 2.0.96 | Sample size calculation |
| broom 1.0.13, broom.mixed 0.2.9.7 | Extraction of model estimates |
| tidyverse 2.0.0 (dplyr 1.2.1, ggplot2 4.0.3, tidyr 1.3.2, readr 2.2.0, stringr 1.6.0, forcats 1.0.1, purrr 1.2.2, tibble 3.3.1), lubridate 1.9.5, janitor 2.2.1, AMR 3.0.1, here 1.0.2 | Data management, age grouping, and figures |
| robotoolbox 1.6.2, dm 1.1.2, haven 2.5.5, readxl 1.5.0 | Import of the KoboToolbox interview data and registry files, and filtering of linked tables |
| gt 1.3.0, patchwork 1.3.2, scales 1.4.0 | Tables and figures |

**Data preparation**

Interview data were downloaded from KoboToolbox and stored as a frozen snapshot. Three submissions that duplicated or misattributed another patient (with their household rosters) and six interviews recorded as refused (consent No on the interview form) were removed before analysis, leaving 669 interviewed patients (307 DTC, 362 HBC), who form the analysis population for every model below unless stated. The sampling frame was matched exactly with MatchIt (method = "exact") on age group (0 to 5, 6 to 10, 11 to 15, and 16 years or more), sex, ward of residence, and registry vaccination status, which gave 143 variable-size subclasses among the interviewed patients.

**Model specifications**

**Mortality (all models fitted to the 669 patients, 24 deaths, unless stated)**

**Primary model (Model A).** Conditional logistic regression, died ~ treatment + age + vaccinated + delay + strata(subclass), exact partial likelihood. The estimate is the conditional odds ratio (OR) for HBC versus DTC.

**Mediator-adjusted model (Model B).** Model A plus clinical complication.

**Exclusion of complications.** Model A refitted without the eight patients with complications (661 patients, 21 deaths).

**Calendar time.** Model A plus a restricted cubic spline of the date of notification (4 knots, 3 degrees of freedom), compared with Model A by likelihood ratio test. An early and late phase of the epidemic (split at the median notification date) was examined with died ~ treatment * phase + age + vaccinated + delay (4 days or more) + strata(subclass).

**Linearity and effect modification of delay.** Logistic regression (rms::lrm) of died on treatment, age, vaccinated, and delay as a restricted cubic spline (4 knots), compared with the linear term by likelihood ratio test. Effect modification: Model A with a treatment by delay (days) interaction, compared with Model A by likelihood ratio test.

**LGA.** Model A plus LGA as a fixed covariate; and an unconditional logistic regression, died ~ treatment + age + vaccinated + delay + (1 | LGA), with the bobyqa optimiser.

**Firth model.** Firth penalised logistic regression, died ~ treatment + age + vaccinated + delay, without conditioning on subclass.

**Vaccination definitions.** Model A with the interview-reported vaccination status replaced by each of the two card-based definitions, keeping the subclasses.

**Composite outcome.** Model A with death or long-term sequelae as the outcome, and the same Firth model.

**Design-based risk ratios.** survey::svydesign(ids = ~1, strata = ~subclass) with quasi-Poisson regression (log link): died ~ treatment (unadjusted) and died ~ treatment + age + vaccinated + delay (adjusted). Single-patient strata were handled with the survey package option survey.lonely.psu = "adjust". Case fatality risks were design-based means by arm.

**Inverse probability of treatment weighting (IPTW).** A logistic regression of DTC allocation on age, age squared, sex, vaccinated, LGA, and household size gave propensity scores. Stabilised weights were trimmed at the 1st and 99th percentiles. The marginal OR came from a weighted quasi-binomial regression of died on treatment; the doubly robust OR added age, vaccinated, and delay. The marginal risk ratio used a weighted quasi-Poisson regression (log link) and the risk difference a weighted Gaussian regression (identity link). Standard errors were HC1 and ignore the estimation of the weights.

**Selection weights.** A logistic regression of interview status among the 986 originally sampled patients on sex, age group, vaccination status at registration, and admission location. Weights were the marginal probability of interview divided by each patient's predicted probability; replacement patients took the probability predicted from their own registry values. The combined weight was the trimmed IPTW weight multiplied by the selection weight, in weighted quasi-binomial regressions of died on treatment, with and without age, vaccinated, and delay.

**Originally sampled patients only.** Model A, the Firth model, and IPTW (propensity model re-estimated) restricted to the 491 originally sampled patients who were interviewed.

**E-values.** EValue::evalues.OR(est, lo, hi, rare = TRUE), applied to the Model A estimates for treatment, vaccination, and delay, with each OR expressed as greater than 1.

**Diagnostics.** An unconditional logistic regression with the Model A covariates gave variance inflation factors (car::vif), the c-statistic (rank-based area under the curve), and observed and predicted mortality by quintile of predicted risk. The within-subclass concordance came from survival::concordance applied to Model A. Influence was assessed with dfbeta residuals from an Efron-method conditional logistic regression with the same covariates, because dfbeta residuals are not available for the exact method.

**Long-term sequelae (645 survivors, 14 with sequelae)**

**Primary.** Conditional logistic regression, sequelae ~ treatment + age + vaccinated + delay + strata(subclass), exact partial likelihood.

**Sensitivity analyses.** The conditional model with treatment alone; Firth penalised logistic regression with treatment alone, with the Model A covariates, and with clinical complication added; the conditional model restricted to the 640 survivors without complications; and design-based risk ratios (quasi-Poisson, log link) and risk differences (Gaussian, identity link) with subclass as stratum.

**Household transmission (household members of the 669 patients)**

**Windows.** Cases were household members with onset 2 to 14 days after the index patient's symptom onset (primary), 2 to 30 days, or at any time.

**Logistic model.** A logistic regression of secondary infection on index treatment, index clinical complication, index delay (4 days or more), index vaccinated, household member's age group, sex, and vaccination status, and household size, with standard errors clustered by household (sandwich::vcovCL). Members with missing covariates, and those who became cases outside the window, were excluded; the model was repeated in the two wider windows.

**Risk ratios and random intercept.** The same covariates in a Poisson regression with cluster-robust standard errors gave risk ratios. A logistic mixed model with a household random intercept, bobyqa (Nelder-Mead if singular or not converged), omitted the member's age group and sex because the full model was singular.

**Acceptability (patient-reported, five-point Likert scales)**

**Linear mixed models.** score ~ treatment + delay (4 days or more) + complication + (1 | subclass), fitted for acceptability, quality, perceived improvement, and their mean (lmerTest, Satterthwaite degrees of freedom). Coefficients are mean differences in Likert points, HBC minus DTC.

**Cumulative-link models.** ordinal::clm with the same fixed effects and no random intercept, for each dimension. A cumulative-link mixed model (clmm) estimated the subclass standard deviation close to zero. CIs for the treatment and delay coefficients came from resampling subclasses with replacement (2,000 replicates, percentile intervals, random seed 2026); replicates that did not meet the convergence criteria were excluded.
