# Supplementary material for: Outcomes of home-based versus facility-based care for mild diphtheria during a large epidemic in Kano State, Nigeria: A retrospective matched cohort study
Source: PLoS Med. 2026 Oct 26;23(10):e1005097. doi: 10.1371/journal.pmed.1005097 (PMC13722324; doi:10.1371/journal.pmed.1005097)
Supplement: Supplementary file 7 [file pmed.1005097.s007.docx]

**S1 Table**

Complete cases and events (or secondary cases) included in each model

| Model | N | Events / cases |
| --- | --- | --- |
| Primary mortality (Model A) | 669 | 24 |
| Complications sensitivity (Model B) | 669 | 24 |
| Complication-exclusion sensitivity | 661 | 21 |
| Firth penalised mortality (sensitivity) | 669 | 24 |
| Calendar-time model | 669 | 24 |
| IPTW / selection-weighted | 669 | 24 |
| Mortality: first card-based vaccination definition (primary model) | 648 | 24 |
| LGA fixed-effect / random-intercept sensitivity | 669 | 24 |
| Mortality: second card-based vaccination definition (primary model) | 275 | 17 |
| Mortality: originally sampled patients only (primary model, Firth, IPTW) | 491 | 15 |
| Mortality: treatment-by-delay interaction | 669 | 24 |
| Composite of death or long-term sequelae (primary model, Firth) | 669 | 38 |
| Long-term sequelae among survivors (primary model and sensitivity models) | 645 | 14 |
| Long-term sequelae among survivors without complications | 640 | 12 |
| Household SAR (primary window, multivariable/multilevel) | 4728 | 136 |
| Household SAR (2 to 30 day window sensitivity) | 4759 | 167 |
| Household SAR (unrestricted window sensitivity) | 4979 | 387 |
| Acceptability (ordinal): Acceptability | 669 | - |
| Acceptability (ordinal): Quality | 668 | - |
| Acceptability (ordinal): Perceived improvement | 669 | - |
