# Supplementary material for: Outcomes of home-based versus facility-based care for mild diphtheria during a large epidemic in Kano State, Nigeria: A retrospective matched cohort study
Source: PLoS Med. 2026 Oct 26;23(10):e1005097. doi: 10.1371/journal.pmed.1005097 (PMC13722324; doi:10.1371/journal.pmed.1005097)
Supplement: Supplementary file 8 [file pmed.1005097.s008.docx]

**S2 Table**

Diagnostics for the primary mortality model (Model A): collinearity, discrimination, calibration, and influence, with the model each comes from

| Metric | Value |
| --- | --- |
| Variance inflation factor (unconditional logistic regression) | |
| Treatment modality (HBC vs DTC) | 1·00 |
| Age | 1·20 |
| Vaccination status | 1·01 |
| Treatment delay | 1·20 |
| Discrimination | |
| c-statistic (area under the receiver operating characteristic curve), unconditional logistic regression | 0·832 |
| Concordance (SE), conditional model within matched subclasses | 0·772 (0·070) |
| Calibration by predicted-risk quintile (unconditional logistic regression) | |
| Quintile 1 (n=134) | Predicted 0·002, observed 0·000 |
| Quintile 2 (n=134) | Predicted 0·008, observed 0·007 |
| Quintile 3 (n=134) | Predicted 0·016, observed 0·022 |
| Quintile 4 (n=134) | Predicted 0·035, observed 0·022 |
| Quintile 5 (n=133) | Predicted 0·120, observed 0·128 |
| Case-level influence (Efron-method refit of the conditional model) | |
| Largest absolute dfbeta, HBC coefficient (log-odds scale) | 0·22 |
