# Supplementary material for: Outcomes of home-based versus facility-based care for mild diphtheria during a large epidemic in Kano State, Nigeria: A retrospective matched cohort study
Source: PLoS Med. 2026 Oct 26;23(10):e1005097. doi: 10.1371/journal.pmed.1005097 (PMC13722324; doi:10.1371/journal.pmed.1005097)
Supplement: Supplementary file 9 [file pmed.1005097.s009.docx]

**S3 Table**

Adjusted odds ratios and risk ratios, household secondary attack, multivariable model (primary 2 to 14 day window)

| Risk factor | Adjusted OR (95% CI) | Risk ratio (95% CI) |
| --- | --- | --- |
| Treatment: HBC vs DTC | 0·71 [0·44,1·16] | 0·72 [0·45,1·15] |
| Complication present | 1·67 [0·68,4·14] | 1·61 [0·70,3·67] |
| Treatment delay ≥4 days | 1·60 [0·96,2·69] | 1·57 [0·96,2·57] |
| Index case vaccinated | 0·68 [0·41,1·12] | 0·69 [0·43,1·11] |
| Contact age 5 to 14 years (vs 0 to 4 years) | 2·28 [1·16,4·49] | 2·21 [1·15,4·27] |
| Contact age 15 years or more (vs 0 to 4 years) | 1·17 [0·59,2·34] | 1·17 [0·60,2·29] |
| Contact sex: male | 0·76 [0·53,1·09] | 0·77 [0·54,1·09] |
| Household size | 0·97 [0·92,1·02] | 0·97 [0·92,1·02] |
| Contact vaccinated | 1·78 [1·15,2·77] | 1·75 [1·14,2·68] |
