# Supplementary material for: Outcomes of home-based versus facility-based care for mild diphtheria during a large epidemic in Kano State, Nigeria: A retrospective matched cohort study
Source: PLoS Med. 2026 Oct 26;23(10):e1005097. doi: 10.1371/journal.pmed.1005097 (PMC13722324; doi:10.1371/journal.pmed.1005097)
Supplement: Supplementary file 10 [file pmed.1005097.s010.docx]

**S4 Table**

Propensity score overlap, covariate balance, effective sample size, and trimming sensitivity

| Metric | Value |
| --- | --- |
| Propensity score | |
| Range (both arms) | 0·248 to 0·794 |
| Proportion of propensity scores <0·05 or >0·95 | 0·0% |
| Patients outside common support | 3/669 (0·4%) |
| Covariate balance (standardised mean difference, raw → weighted) | |
| Age | 0·032 → 0·004 |
| Vaccinated | 0·088 → 0·008 |
| Household size | 0·191 → 0·015 |
| Effective sample size (untrimmed → trimmed) | |
| DTC (n=307) | 302 → 302 |
| HBC (n=362) | 354 → 357 |
| Trimming sensitivity (HBC vs DTC OR) | |
| Untrimmed | 0·29 [0·12,0·72] |
| 5th/95th percentile | 0·30 [0·12,0·74] |
| 1st/99th percentile (primary) | 0·29 [0·12,0·72] |
