# Supplementary material for: Outcomes of home-based versus facility-based care for mild diphtheria during a large epidemic in Kano State, Nigeria: A retrospective matched cohort study
Source: PLoS Med. 2026 Oct 26;23(10):e1005097. doi: 10.1371/journal.pmed.1005097 (PMC13722324; doi:10.1371/journal.pmed.1005097)
Supplement: Supplementary file 11 [file pmed.1005097.s011.docx]

**S5 Table**

Covariate balance between arms among the 669 interviewed patients, including replacements, and comparison of interviewed and not-interviewed patients among the 986 originally sampled

| Metric | Value |
| --- | --- |
| Standardised mean difference, HBC minus DTC, variables matched on (669 interviewed patients) | |
| Sex: Male | 0·039 |
| Sex: Female | -0·039 |
| Age group: ≥16 years | -0·035 |
| Age group: 6 to 10 years | -0·012 |
| Age group: 11 to 15 years | 0·024 |
| Age group: 0 to 5 years | 0·028 |
| Vaccination status at registration: Not vaccinated | -0·104 |
| Vaccination status at registration: Fully vaccinated | 0·096 |
| Vaccination status at registration: Partially vaccinated | 0·029 |
| LGA: Nassarawa | -0·008 |
| LGA: Fagge | 0·028 |
| LGA: Ungogo | -0·059 |
| LGA: Dawakin Tofa | 0·093 |
| Standardised mean difference, HBC minus DTC, other baseline variables (669 interviewed patients) | |
| Household size | -0·191 |
| Comorbidity | -0·182 |
| Complication | -0·231 |
| Interviewed vs not interviewed (986 originally sampled patients) | |
| Treatment setting: DTC | interviewed=250, not interviewed=246 (p=0·750) |
| Treatment setting: HBC | interviewed=241, not interviewed=249 (p=0·750) |
| Sex: Male | interviewed=180, not interviewed=148 (p=0·029) |
| Sex: Female | interviewed=311, not interviewed=347 (p=0·029) |
| Age group: 0 to 5 years | interviewed=72, not interviewed=63 (p=0·136) |
| Age group: 6 to 10 years | interviewed=201, not interviewed=178 (p=0·136) |
| Age group: 11 to 15 years | interviewed=100, not interviewed=106 (p=0·136) |
| Age group: ≥16 years | interviewed=118, not interviewed=148 (p=0·136) |
| Vaccination status at registration: Fully vaccinated | interviewed=115, not interviewed=93 (p=0·043) |
| Vaccination status at registration: Not vaccinated | interviewed=371, not interviewed=389 (p=0·043) |
| Vaccination status at registration: Partially vaccinated | interviewed=5, not interviewed=13 (p=0·043) |
