# Supplementary material for: Outcomes of home-based versus facility-based care for mild diphtheria during a large epidemic in Kano State, Nigeria: A retrospective matched cohort study
Source: PLoS Med. 2026 Oct 26;23(10):e1005097. doi: 10.1371/journal.pmed.1005097 (PMC13722324; doi:10.1371/journal.pmed.1005097)
Supplement: Supplementary file 12 [file pmed.1005097.s012.docx]

**S6 Table**

Cumulative-link (proportional-odds) sensitivity models for each Likert dimension

| Term | OR | 95% CI | p (model-based) |
| --- | --- | --- | --- |
| Acceptability | | | |
| Treatment: HBC vs DTC | 0·61 | [0·42,0·87] | 0·007 |
| Treatment delay ≥4 days | 0·58 | [0·40,0·86] | 0·012 |
| Complication present | 0·23 | [0·06,0·92] † | 0·037 |
| Quality | | | |
| Treatment: HBC vs DTC | 0·66 | [0·44,1·00] | 0·030 |
| Treatment delay ≥4 days | 0·58 | [0·40,0·82] | 0·015 |
| Complication present | 0·76 | [0·15,3·86] † | 0·742 |
| Perceived improvement | | | |
| Treatment: HBC vs DTC | 0·93 | [0·57,1·53] | 0·746 |
| Treatment delay ≥4 days | 0·48 | [0·31,0·79] | 0·002 |
| Complication present | 0·25 | [0·07,0·92] † | 0·038 |
| OR = odds ratio for a more favourable response; HBC compared with DTC. CI = confidence interval. CIs for treatment and delay are percentile intervals from 2,000 resamples of the matched subclasses; resamples that did not meet convergence criteria were excluded (acceptability 83 of 2,000; perceived improvement 33 of 2,000; quality 244 of 2,000). † Model-based Wald interval: the complication coefficient was unstable in resamples with few complicated respondents and was not bootstrapped. p-values are model-based Wald p-values. | | | |
